# Study on the mechanism of action of Wu Mei Pill in inhibiting rheumatoid arthritis through TLR4-NF-κB pathway

**DOI:** 10.1186/s13018-024-04551-z

**Published:** 2024-01-13

**Authors:** Yuheng Fu, Chunyu Gao, Xialin Sun, Yan Zhao, Haibo Zhang

**Affiliations:** 1 Tianjin University of Traditional Chinese Medicine, Tianjin, China; 2 College of Chinese Medicinal Materials, Jilin Agricultural University, Changchun, China; 3 College of Pharmacy, Jilin Medical University, Jilin City, China; 4 Changchun University of Chinese Medicine, Changchun, China

**Keywords:** Wu Mei Pill, Rheumatoid arthritis, Fibroblastic synovial cells, Invasion and migration, TLR4-NF-κB pathway

## Abstract

**Background:**

Wu Mei Pills (WMP) is a traditional Chinese medication that exhibits considerable anti-inflammatory effects. While WMP has been documented for its efficacy in treating RA, its mechanism of action on the condition remains unestablished.

**Methods:**

The chemical composition of WMP was analyzed through UPLC-MS. Next, the enzyme-linked immunosorbent assay, cell scratch, Transwell, and Western blotting techniques were used to investigate its intrinsic mechanism. Lastly, the effect of WMP in inhibiting RA was explored by applying it to CIA rats.

**Result:**

UPLC-MS analysis detected 181 compounds in WMP. RA-FLS migration and invasion mechanisms were significantly hindered by serum containing WMP (2%, 8%). Moreover, WMP (0.5 g/kg, 2 g/kg) restricted arthritis and immune organ indices in CIA rats with type II collagen-induced rheumatoid arthritis by blocking TLR4-NF-κB inflammatory pathway activation.

**Conclusions:**

WMP is valuable in mitigating the course of RA through inhibiting the classical TLR4-NF-κB inflammatory pathway and reducing the secretion of inflammatory factors in the serum of RA-FLS and CIA rats. Moreover, it regulates the dynamic balance of MMP-2/TIMP-2, MMP-9/TIMP-1, modulates the mechanism of RA-FLS invasion, and safeguards articular cartilage tissues in RA.

**Supplementary Information:**

The online version contains supplementary material available at 10.1186/s13018-024-04551-z.

## Introduction

Rheumatoid arthritis (RA) is a condition resulting from autoimmune hyperactivity, characterized by joint inflammation, proliferation of synovial tissue, and angiogenesis. Prolonged RA can result in disability and adversely affect an individual's quality of life [1]. The statistics indicate that approximately 1% of the global population is affected by RA. The pathogenesis is intricate and influenced by genetic and environmental factors, along with various other determinants. Moreover, research on the pathogenesis of RA is still in its exploratory phase [2].

Research has identified pattern recognition receptors (PRRs) as a significant contributor to the pathogenesis of RA. The Toll-like receptor (TLR) family comprises the key molecule in pattern recognition receptors (PRRs) and is able to present antigens to effector cells, thereby activating them [3]. As researchers delve deeper into this area, they have identified around 13 receptors within the Toll family [4]. TLRs are protein structures that link the inner and outer membranes of a cell, regulating non-specific immunity. Research has demonstrated that when lipopolysaccharide (LPS) binds to TLR4, it leads to an increase in its expression. This, in turn, leads to abnormalities in the expression of associated proteins downstream, triggers a cascade of inflammatory pathways. The resulting effect can exacerbate joint inflammation in patients [5, 6]. Activation of TLR4 further enhances the activation of nuclear factor-κB (NF-κB). The NF-κB pathway plays an equally influential role in promoting the development of RA. Elevated NF-κB protein expression was found in the synovium of RA, which enters the nucleus and contributes to the transcription and secretion of relevant inflammatory factors [7]. The aforementioned inflammatory factors exacerbate inflammation and joint damage, thereby perpetuating the progression of RA [8]. Additionally, an excessive TLRs response induces the uncontrolled release of matrix metalloproteinases (MMPs) [9]. Matrix metalloproteinases are deemed to play a noteworthy role in the degradation of articular cartilage within affected joints [10]. The overexpression of MMPs can result in joint surface and cartilage erosion in RA [11, 12]. Thus, matrix metalloproteinase imbalance may be an important factor contributing to joint destruction in RA.

According to TCM, rheumatoid arthritis is a pathological condition arising from the invasion of external factors, including wind, dampness, cold and other unfavorable environmental factors. Throughout the extensive history of Chinese medicine’s treatment of RA, traditional Chinese medicine has extensively employed herbs, whether in compound formulas or single-flavored preparations, which function to dispel wind, cold, and dampness from the body. Several studies have shown that when DMARDs are used in conjunction with Chinese herbal medicines, the drugs are significantly more effective in relieving RA symptoms and reducing associated side effects. In recent years, therapeutic drugs targeting immune cells and related cytokines (e.g. TNF-α, IL-6, etc.) have also achieved excellent clinical efficacy [13, 14]. Although there have been advancements in treating RA, some patients experience swift joint destruction while under treatment. However, the clinical options are also limited due to drugs' side effects, such as liver and kidney damage, immunosuppression, and the high cost of biologics. As a result, the quest for new anti-rheumatoid arthritis drugs from traditional Chinese medicine could become a new approach [15].

The Wu Mei Pill (WMP) is a traditional Chinese compound medicine dating back to the Han Dynasty. This medicine is primarily composed of 16 traditional Chinese herbs, including dark plum, Chinese prickly ash, Asarum and Coptic chinensis. Previous studies have shown that WMP is able to regulate the body's immunity and has an inhibitory effect on inflammation [16, 17]. Relevant research has demonstrated that WMP effectively reduces serum levels of inflammatory factors and inhibits the overactivation of the TLR4-MyD88-NF-κB inflammatory pathway in mice exhibiting intestinal mucositis. Moreover, WMP has been shown to restore balance to intestinal flora and alleviate intestinal mucosal inflammation in mice [18]. Furthermore, WMP inhibits macrophage activation by impacting the Notch/NF-κB/NLRP3 pathway. It also exerts an inhibitory effect on immune overactivation and anti-inflammatory bursts within the body [19]. In summary, scientific studies confirm that WMP exhibits substantial anti-inflammatory properties and shown to regulate key proteins related to inflammation, such as TLR4 and NF-κB, which are closely associated with RA pathogenesis. According to the traditional Chinese medicine theory, WMP is primarily administered to address various ailments triggered by cold. RA is classified as a “Bi” syndrome caused by cold, wind, and dampness, and is within the therapeutic domain of WMP in ancient Chinese medicine. Consequently, this experiment primarily explored the specific mechanism through which WMP alleviates the progression of RA.

## Result

### Identification of the chemical composition of WMP

**Fig. 1:**
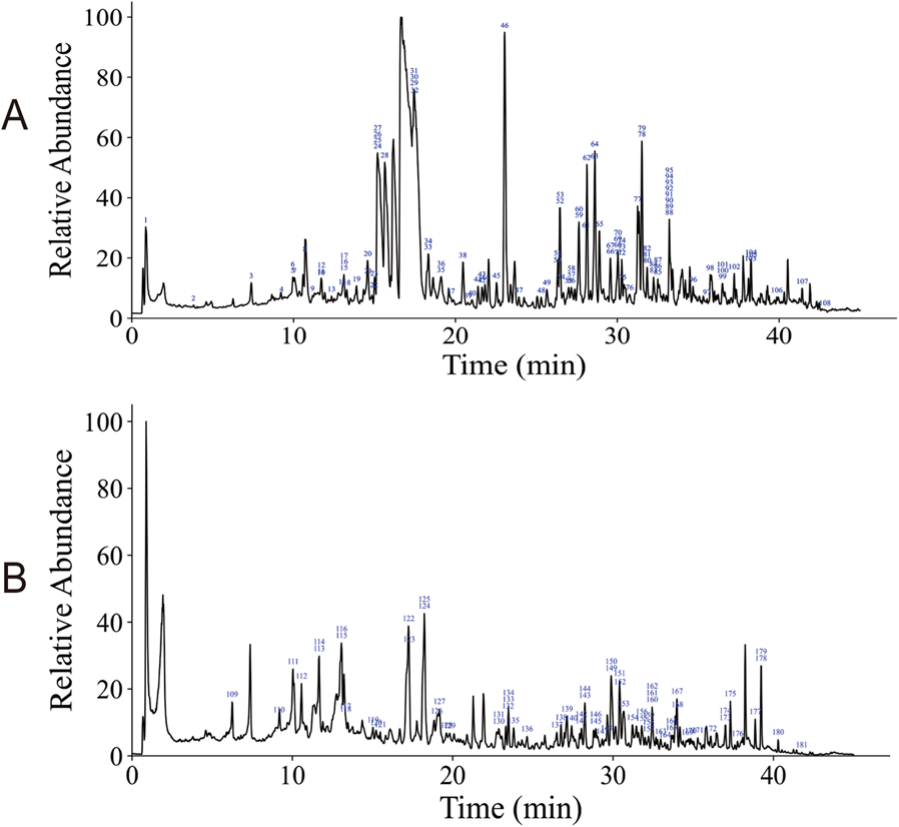
Total ion chromatogram of constituents in WMP. **A** Positive ion mode. **B** Negative ion mode

A total of 181 compounds were detected in the WMP in both positive (Fig. 1A) and negative (Fig. 1B) ionization modes. Additional file 1: Table S1 lists the compounds contained in each herb. Wumeirou comprises of 2 compounds, Xixin comprises of 2 compounds, Huanglian comprises of 3 compounds, Huangbai comprises of 1 compound, Ganjiang comprises of 51 compounds, Heishunpian comprises of 4 compounds, Guizhi comprises of 2 compounds, Renshen comprises of 6 compounds, Danggui comprises of 29 compounds, Zhike comprises of 4 compounds, Jiegeng comprises of 1 compound, Baishao comprises of 16 compounds, and Zhigancao comprises of 74 compounds. The entire list of 181 compounds can be found as Additional file 1: Table S1 online. Furthermore, we have identified every herb based on its markers to verify the precision of the herbs employed. The fingerprints for specific experiments can be found as Additional file 1: Fig. S2 online.

**Table 1:** Herbal constituents of WMP

Chinese name	Latin name	Dose (g)
Wumei	*Prunus mume* (Siebold) Siebold & Zucc	120
Huajiao	*Zanthoxylum bungeanum* Maxim	12
Xixin	*Asarum heterotropoides* F.Schmidt	18
Huanglian	*Coptis omeiensis* (F.H.Chen) C.Y.Cheng	48
Huangbai	*Phellodendron chinense* C.K.Schneid	18
Ganjiang	*Zingiber officinale* Roscoe	30
Heishunpian	*Aconitum carmichaelii* Debeaux	18
Guizhi	*Neolitsea cassia* (L.) Kosterm	18
Renshen	*Panax ginseng* C.A.Mey	18
Danggui	*Angelica sinensis* (Oliv.) Diels	12
Zhike	*Citrus* × *aurantium* L.	12
Jiegeng	*Platycodon grandiflorus* (Jacq.) A.DC	12
Baishao	*Paeonia lactiflora* Pall	12
Zhigancao	*Glycyrrhiza glabra* L.	12

### Effect of WMP on the activity and inflammatory factors of RA-FLS cells

**Fig. 2:**
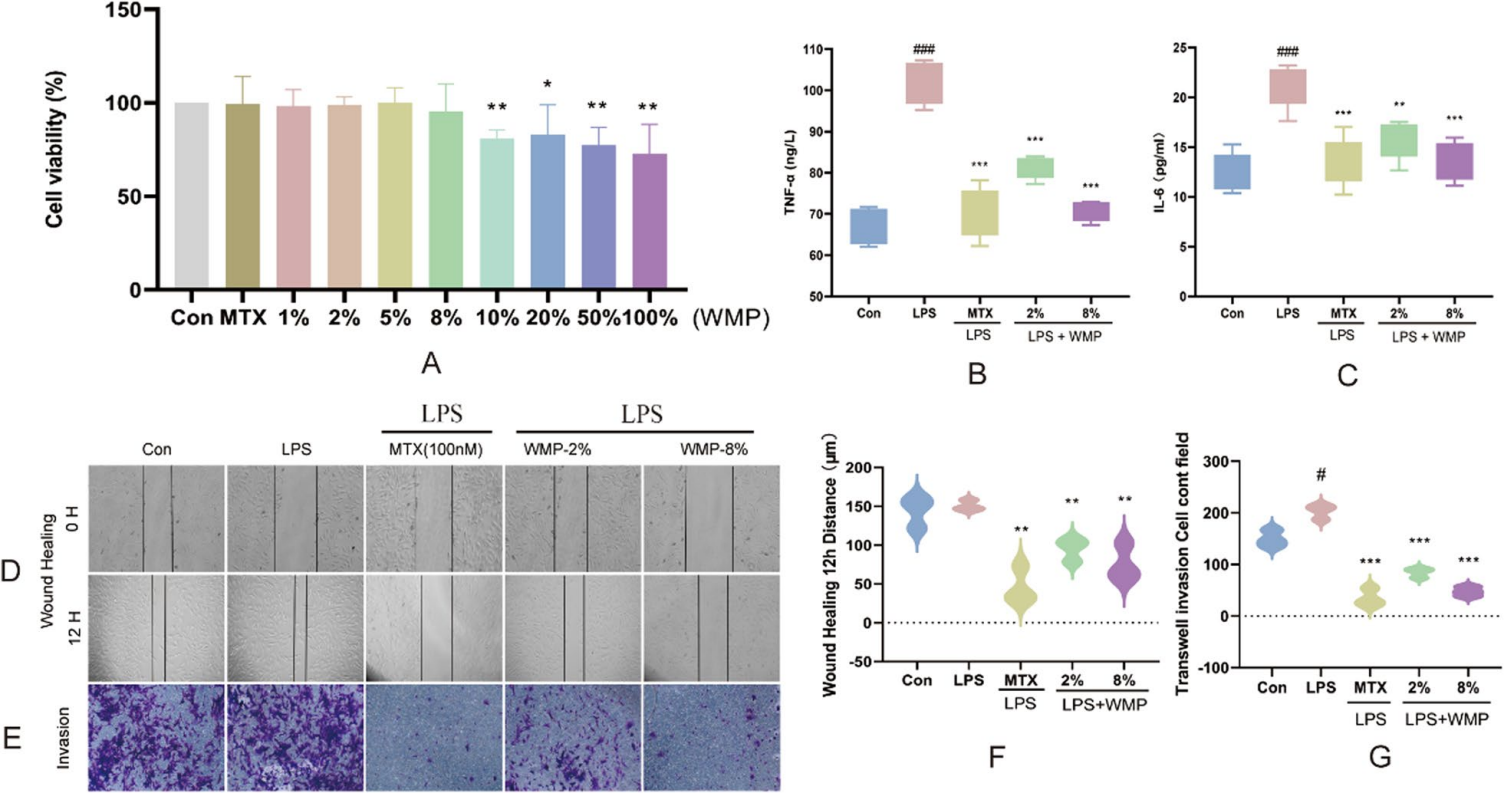
WMP decreased RA-FSL viability migration and invasion. **A** Cell viability assay by CCK-8 assay. **B** and **C** show the levels of TNF-α, IL-6 in RA-FSL in each group by ELISA. The **D** and **E** are WMP inhibition of RA-FLS migration and invasion (magnification × 400). The **F** and **G** are data statistics Data are expressed as mean ± standard deviation. (#) *p* < 0.05, (##) *p* < 0.01 and (###) *p* < 0.001 versus control. (*) *p* < 0.05, (**) *p* < 0.01 and (***) *p* < 0.001 versus LPS

The dose-dependent impact of WMP on RA-FLS activity is illustrated in Fig. 2A. Our research objectives were twofold: to investigate the mechanisms underlying WMP's anti-inflammatory properties and its influence on RA-FLS migration and invasion. Cell Counting Kit-8 (CCK-8) tests showed no significant effect on the viability of RA-FLS when treated with 2% or 8% WMP, or 100 nM MTX. Therefore, these experimental doses were used for subsequent migration and invasion assay experiments, as well as for studying mechanisms related to anti-inflammatory activity.

Based on the ELISA results, it was found that LPS had a significant impact on increasing the levels of TNF-α and IL-6 in RA-FLS cells (*p* < 0.01). This suggests that RA-FLS cells remained activated in an inflammatory environment. Conversely, Fig. 2B and C showed that WMP significantly inhibited the secretion of both TNF-α (*p* < 0.001) and IL-6 (*p* < 0.001). This implies that WMP has a significant anti-RA inflammatory effect.

### Effect of WMP on RA-FLS migration and invasion

In the wound healing assay, Fig. 2D and F depict considerable migration of RA-FLS in both the control and LPS groups, with scratches nearly disappearing within 12 h. However, the horizontal migration of RA-FLS was significantly inhibited by application of MTX (100 nM) and WMP (2%, 8%) (*p* < 0.01). As evidenced in Fig. 2E and G, the vertical invasion of RA-FLS was significantly inhibited by MTX (100 nM) and WMP (2%, 8%) in the transwell assay (*p* < 0.05 and *p* < 0.001, respectively).

### Effect of WMP on the expression of inflammatory related proteins in RA-FLS

**Fig. 3:**
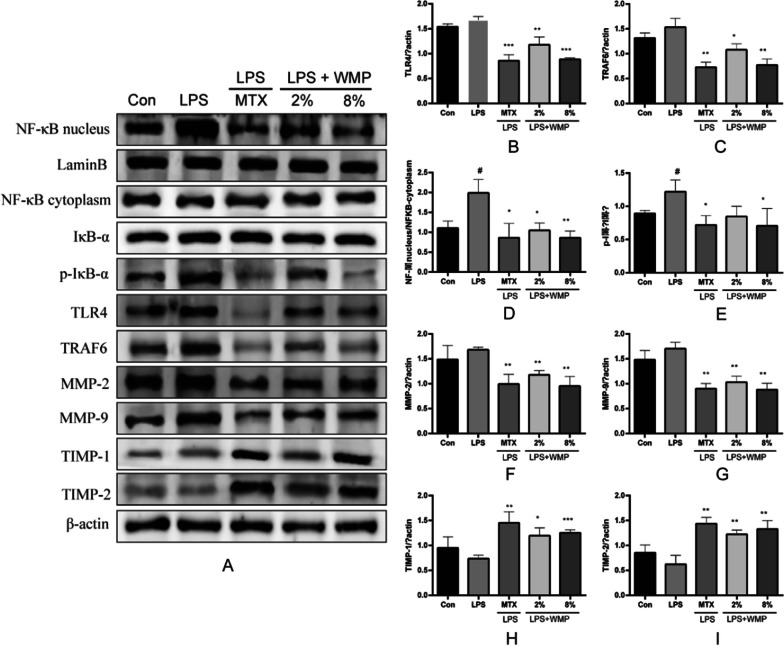
WMP reduces RA-FLS inflammation by inhibiting the TLR4/NF-κB signaling pathway and decreases RA-FLS migration and invasion by inhibiting MMPs. **A** depicts the western blot illustrating the impact of WMP on the expression of RA-FLS proteins in each group. **B**–**I** depict the expression levels of TLR4, TRAF6, NF-κB, IκB-α, MMP-2, MMP-9, TIMP-1, and TIMP-2 proteins in each group of RA-FLS. Data are expressed as mean ± standard deviation (*n* = 3). (#) *p* < 0.05, (##) *p* < 0.01 and (###) *p* < 0.001 versus control. (*) *p* < 0.05, (**) *p* < 0.01 and (***) *p* < 0.001 versus LPS

The TLR4/NF-κB pathway is a subject of frequent study in the context of RA. We scrutinized the effects of WMP on this particular pathway in RA-FLS cells through protein immunoblotting. In contrast to the control group, the LPS group exhibited elevated levels of inflammation-related protein expression, signifying a further exacerbation of RA-FLS inflammation. As demonstrated in Fig. 3B–E of the experimental results, the use of WMP yielded significant inhibitory effects on TLR4 protein expression (*p* < 0.001), TRAF6 protein expression (*p* < 0.01), nuclear translocation of NF-κB proteins (*p* < 0.01), and the phosphorylation of IκB-α (*p* < 0.05), in comparison to the LPS group. Furthermore, the experimental results Fig. 3F–I demonstrate that WMP significantly hindered the expression of MMP-2/9 protein in RA-FLS cells (*p* < 0.01) and encouraged the expression of TIMP-1/2 protein in RA-FLS cells compared to the LPS group (*p* < 0.001, *p* < 0.01).

### Effect of WMP on arthritis index, immune organ index and inflammatory factors in CIA rats

**Fig. 4:**
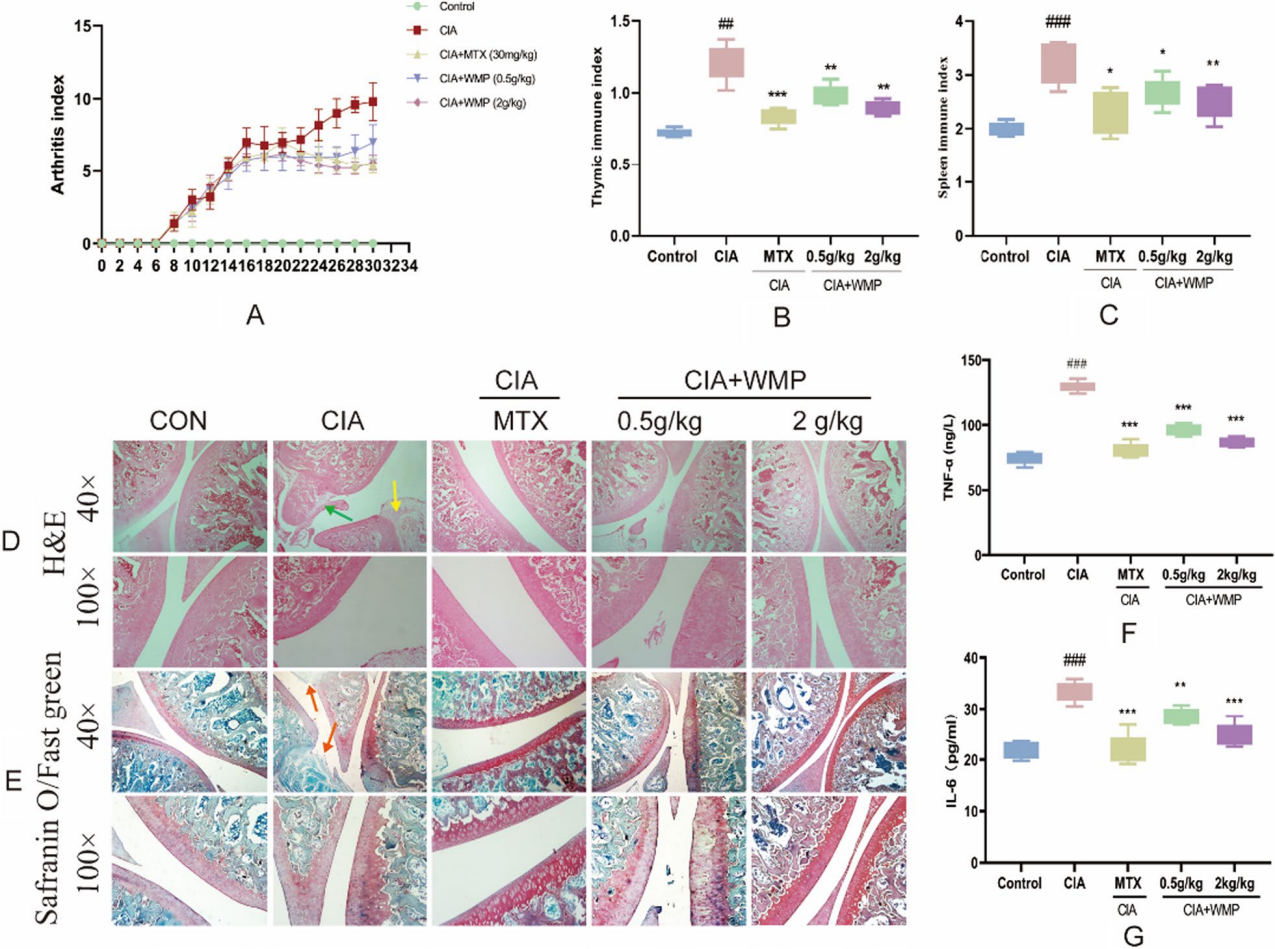
WMP significantly reduced joint inflammation in CIA rats. **A** indicates the arthritis index score of rats in each group. **B** and **C** display the spleen and thymus immunity index of CIA rats in each group. **D** indicates H&E staining (green arrow indicates synovial hyperplasia, yellow arrow indicates inflammatory infiltrate). **E** indicates safranin O/fast green staining (orange arrow indicates cartilage band erosion). **F** and **G** depict the expression of TNF-α and IL-6 in the serum of rats in each group. Data are expressed as mean ± standard deviation (*n* = 5). (#) *p* < 0.05, (##) *p* < 0.01 and (###) *p* < 0.001 versus control. (*) *p* < 0.05, (**) *p* < 0.01 and (***) *p* < 0.001 versus CIA

In this experiment, while performing the modeling, and WMP administration, the arthritis index scores of rats in each group were performed every 2 days, and the results are shown in the results of Fig. 4A. Except for the control group, the rats in the remaining groups reached a score of 6 or more around day 15, and WMP administration was started. The arthritis index of the WMP (2 g/kg) group was significantly suppressed until the 30th day.

Figure 4B and C demonstrates a marked inflammatory response in rats following collagen induction, as indicated by the elevated ratio of immune organs (thymus, spleen) to body weight, which was significantly greater than that of the control group (*p* < 0.01). Positive drug and WMP administration began early in the model stage. After one cycle of treatment, inflammation reduced in rats. Evidence of this is shown by a decrease in immune organ ratios (thymus, spleen) to body weight in CIA rats who received WMP treatment, which was significantly different from the model group (*p* < 0.01 and *p* < 0.05, respectively). This indicates that when compared to the hyperimmunity of RA rats, WMP effectively reduces it.

In this research, we evaluated the level of inflammation in rat serum. Figure 4F and G demonstrates a substantial increase in levels of both TNF-α and IL-6 in the serum of rats with CIA, providing evidence of elevated autoinflammation. Our findings were consistent with the immune index analysis. Comparatively, rats in the WMP group had significantly reduced levels of TNF-α and IL-6 when compared with the CIA group (*p* < 0.001, *p* < 0.001 respectively). This supports our conclusion that WMP has a significant anti-inflammatory effect on RA rats in vivo.

### Effect of WMP on knee joint swelling in CIA rats

As depicted in Fig. 4D, examination of the knee joint pathological sections revealed that the RA model rats induced by collagen exhibited joint irregularities and showed evident cartilage erosion compared to the healthy rats. This was accompanied by thickening of synovial tissue and inflammatory infiltration. However, after treatment with WMP, the articular surface appeared notably intact, and the inflammatory infiltration was less prominent.

The articular cartilage structure in the knee and ankle joints of rats from each group was examined using safranin O/fast green staining. Figure 4E demonstrates that the surface of the knee joints of the rats in the control group was smooth, and the cartilage (red part) bands were firmly attached to the bone surface. The rats in the CIA group showed varying degrees of joint damage, including cartilage thinning or disappearance, as well as areas of severe cartilage destruction. Nevertheless, following WMP treatment, the cartilage bands appeared smooth and complete, and the bone tissue structure remained intact. In addition, synovial hyperplasia was relieved, and no obvious inflammatory infiltration was observed.

### Effect of WMP on the expression of inflammatory and invasive proteins in the synovium of CIA rats

**Fig. 5:**
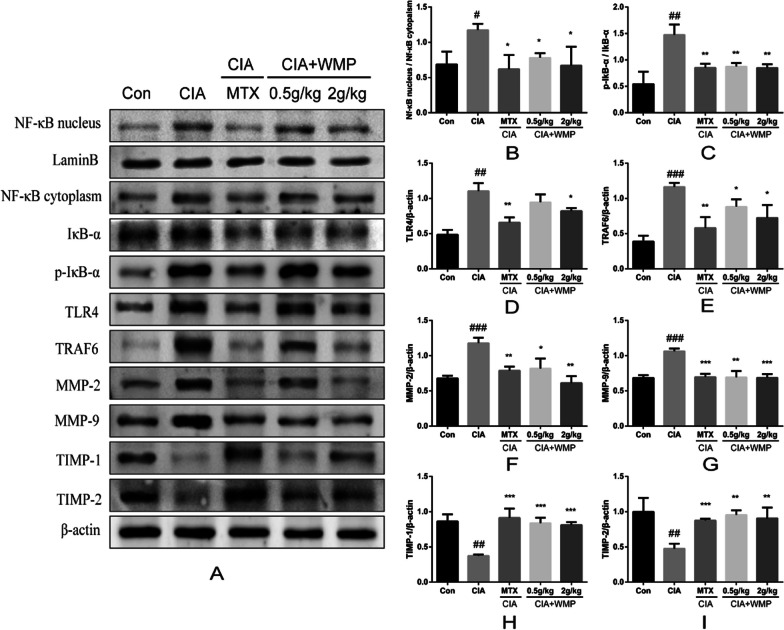
WMP reduces inflammation in CIA rats by inhibiting the TLR4/NF-κB signaling pathway and reduces cartilage invasion by decreasing MMPs. **A** indicates western blot of WMP-affected synovial membrane-associated protein expression in CIA rats. **B**–**I** shows the levels of TLR4, TRAF6, NF-κB, IκB-α, MMP-2, MMP-9, TIMP-1, and TIMP-2 protein expression in the synovial membrane of each group. Data are expressed as mean ± standard deviation (*n* = 3). (#) *p* < 0.05, (##) *p* < 0.01 and (###) *p* < 0.001 versus control. (*) *p* < 0.05, (**) *p* < 0.01 and (***) *p* < 0.001 versus CIA

The proteins related to the synovium in rats with collagen-induced arthritis (CIA) were analyzed through Western blotting. Figure 5B–E illustrates a significant increase in the expression levels of TLR4 and TRAF6 proteins in the CIA group (*p* < 0.01 and *p* < 0.001, respectively). The CIA group also experienced marked nuclear translocation of NF-κB (*p* < 0.05) and phosphorylation of IκB-α (*p* < 0.001). The study's results indicate that WMP significantly hindered the expression of TLR4 and TRAF6 proteins compared to CIA (*p* < 0.05). In addition, nuclear translocation of NF-κB was significantly inhibited (*p* < 0.05), as was phosphorylation of IκB-α (*p* < 0.01). In the study of migration and invasion mechanisms, a significant increase in MMP-2/9 expression in the CIA group (*p* < 0.001) was shown to be associated with synovial migration and invasion according to the results F–I. In contrast, the expression of TIMP-1/2, which prevents synovial migration and invasion by binding to MMP-2/9, was dramatically decreased (*p* < 0.01). In addition, after WMP treatment, excess free MMP-2/9 protein was inhibited (*p* < 0.01, *p* < 0.001) and TIMP-1/2 expression was elevated ((*p* < 0.001, *p* < 0.01), inhibiting synovial inflammation from invading periarticular tissues and cartilage.

## Discussion

WMP is best described in the Han Dynasty Chinese Treatise on *Typhoid Fever*. It primarily treats a variety of cold and heat-induced ailments in Traditional Chinese Medicine theory. The theory posits that RA is mainly caused by external agents like wind, cold, and dampness invading the body. Consequently, the application of WMP in treating RA aligns perfectly with Traditional Chinese Medicine theory. Additionally, recent medical research has indicated a positive anti-inflammatory effect of WMP. Furthermore, scholars have utilized WMP in the treatment of RA, although the exact mechanisms of action remain unclear. Hence, this paper aims to explore the mechanism of WMP in the prevention and management of RA. Ultra performance liquid chromatography-tandem mass spectrometry (UPLC-MS) analysis revealed that WMP contains 181 chemical components. Wumeirou has 2 compounds, Xixin has 2 compounds, Huanglian has 3 compounds, Huangbai has 1 compound, Ganjiang has 51 compounds, Heishunpian has 4 compounds, Guizhi has 2 compounds, Renshen has 6 compounds, Danggui has 29 compounds, Zhike has 4 compounds, Jiegeng has 1 compound, Baishao has 16 compounds, and Zhigancao has 74 compounds. Among the compounds detected in WMP, some have been confirmed to possess significant anti-inflammatory and antioxidant activity. For instance, Neochlorogenic acid in Wumeirou mitigates hepatic lipid accumulation and inflammation by modulating miR-34a [20]. Coptisine in Huanglian can be used to treat NLRP3 inflammasome-mediated gouty arthritis by inhibiting caspase-1 to block NLRP3 inflammasome activation [21]. Zingerone in dried ginger attenuates Ti particle-induced inflammatory osteolysis by inhibiting the NF-κB signaling pathway in osteoclasts [22].

RA is a multifactorial and complex immune-mediated disease with an unclear pathogenesis. Clinical treatment aims to reduce disease activity and alleviate symptoms. Studies suggest that the immune-inflammatory response plays a crucial role in the development of RA and is continuously activated throughout the disease process [23]. TLR4 receptors are transmembrane proteins situated on the cell membrane, which play a critical role in non-specific immunity and can trigger inflammation. Numerous studies support the notion that activation of TLR4 can worsen RA symptoms. Thus, exploring this receptor's properties and analyzing its corresponding pathways could serve as a basis for elucidating RA's pathogenesis [24]. Upon activation by external stimuli, TLR4triggers the secretion of inflammatory factors and chemokines via a MyD88-dependent pathway. Pro-inflammatory factors and chemokines govern intracellular signaling pathways, thereby modulating cellular inflammatory responses in terms of their nature, extent, and timing [25]. Activation of the MyD88-dependent pathway through interference with TLR4 interacts with IRAK, leading to an increase in TRAF6 protein expression [26]. TRAF6 additionally enhances the nuclear translocation of NF-kB, a prominent pro-inflammatory transcription factor, and produces inflammatory factors and mediators [27]. The elevated expression of TLRs in activated FLS has been linked to the activation of the NF-kB pathway and transcription of inflammatory factors like TNF-α and IL-6 [28]. The experiment illustrated that WMP significantly diminished the arthritis and immune organ indices of CIA rats, while also mitigating joint swelling and autoimmune hyperactivation. revealed that WMP notably diminished inflammatory infiltration in the rats' knee joints and conserved cartilage tissue. Furthermore, WMP substantially decreased the levels of TNF-α and IL-6 levels in the CIA rat model. The Western blot analysis results indicate that WMP significantly decreased the expression of TLR4 and TRAF6 proteins. Furthermore, WMP has the ability to suppress the classical NF-κB inflammatory pathway by preventing the nuclear transportation of NF-κB and the phosphorylation of IκB-α.

FLS constitute 70–80% of synovial tissue and serve to uphold the structural integrity of the synovial lining while secreting lubricating fluid. Numerous studies indicate that RA-FLS play a crucial role in the development of RA and are the principal effector cells in the disease. It is important to note that these statements are objective and supported by scientific evidence [29]. The hyperproliferation of RA-FLS, which promotes the production of inflammatory factors and extracellular matrix regulators, has been identified as the primary cause of RA development, leading to joint destruction. Additionally, RA-FLS exhibits tumor-like properties and secretes substantial quantities of MMPs during proliferation, leading to the erosion of cartilage and joint destruction [30]. MMP-2 (also referred to as gelatinase-A) and MMP-9 (also known as gelatinase-B) are both capable of degrading the intercellular matrix, thus promoting the migration and invasion of RA-FLS cells. Subsequently, it is suggested that these MMPs play a role in RA pathogenesis [31, 32]. Significant increases in MMP-2 and MMP-9 proteins have been found to contribute to joint opacification and irreversible cartilage erosion in RA patients, leading to impaired mobility [33]. Under normal circumstances, tissue inhibitors of MMPs (TIMPs) bind to MMPs in a 1:1 ratio, inhibiting the excessive production of MMPs. When rheumatoid arthritis (RA) develops, the balance is disrupted due to the concentration of specific inflammatory factors present in the joints. Therefore, there is an excessive production of MMPs, further accelerating ECM degradation, thereby leading to RA-FLS migration and invasion [34]. TIMP-1 and TIMP-2 bind with MMP-9 and MMP-2 proteins to create a complex and prevent their excessive production [35]. Previous studies have reported elevated MMP expression in the synovial tissue, synovial fluid, and peripheral blood of patients with rheumatoid arthritis [36]. This article examines the mechanism of action of WMP on RA-FLS. Firstly, WMP significantly reduces the secretion of TNF-α and IL-6 inflammatory factors by RA-FLS. Additionally, WMP inhibits RA-FLS migration and invasion significantly through the cell scratch assay and transwell assay. The Western blotting results revealed that WMP substantially reduced the protein expression of MMP-2 and MMP-9 in RA-FLS, increased the protein expression of TIMP-1 and TIMP-2, and rectified the dynamic equilibrium of MMPs' protein expression. This intrinsic mechanism of WMP inhibits RA-FLS's migration and invasion.

In conclusion, WMP demonstrates a significant impact in hindering RA lesions and joint degradation. WMP operates by reducing the levels of TLR4 and TRAF6 proteins, thereby suppressing immune hyperactivity in RA, ultimately impeding the nuclear translocation of NF-κB and IκB-α phosphorylation, thus curtailing the production of inflammatory cytokines (e.g., TNF and IL-6) in RA-α. Additionally, WMP regulates the MMP-2/TIMP-2 and MMP-9/TIMP-1 balance, ultimately hindering RA-FLS migration and invasion. Thus, WMP may hold potential as a future drug for the prevention and treatment of RA.

## Methods

### Drug and drug-containing serum preparation

WMP has been included in the 2020 edition of the Chinese Pharmacopoeia. The study introduced six more Chinese herbal medicines, resulting in a total of 16 Chinese herbal medicines (refer to Table 1).

The drug formulation containing WMP serum: The drug is dissolved in 0.1% sodium carboxymethylcellulose and administered by gavage to mice at a dose of 2 g/kg/day for 7 days. After 15 h of fasting and dehydration, blood is collected and centrifuged to obtain serum. The serum was incubated in a water bath for 25 min to produce drug-containing serum for inactivation. The serum is then filtered through a sterile 0.22 μm microporous membrane under controlled aseptic conditions and stored at − 80 °C. (Note: Different sera can be used. (Note: Different concentrations of drug serum were diluted from normal mouse serum).

### UPLC-MS spectrometry detection

Column: Waters UPLC HSS T3. Mobile phase: methanol, water + 0.1% formic acid. Flow rate: 0.3 ml/min. Sample injection volume: 10 µl. The mass spectrometer was a quadrupole orbital ion trap mass spectrometer (containing a thermospray ion source, Q Active ™) (Table 2).

**Table 2:** Elution gradient table

Time (min)	Mobile phase	
	*A* (v%)	*B* (v%)
0	2	98
1.0	2	98
41.0	100	0
50.0	100	0
50.1	2	98
52.0	2	98

### Establishment of a rheumatoid arthritis model and isolation and cultivation of RA-FLS

The study utilized female Wistar rats (180 ± 20 g, Ease Animal Technology Ltd.), fed a high-fat diet with normal water intake and 12-h cycles of day and night. The Animal Ethics Committee of Jilin Agricultural University approved all experiments.

CAI modeling: 1 ml of bovine type II collagen solution was repeatedly mixed with 1 ml of IFA to make a water-in-oil emulsion. The resulting emulsion was subcutaneously injected into the tails of rats at a dose of 0.2 ml per rat. After 1 week, the same dosage of emulsion was injected again to improve the immunization.

From the first injection of type II collagen to establish a model, the joint swelling of each group of rats is recorded every 2 days. The arthritis index score is a slight adjustment based on previous studies [37]. The score mainly evaluates the joint redness and swelling in rats after the onset of the disease. Each foot is scored out of 4 points, with a total of 16 points. A score of 6 or more indicates successful modeling. The score is based on the degree and extent of joint redness and swelling, as well as joint swelling and deformity. The scoring system is based on a 1–4 point scale, with the following criteria: (1) 0 points: No redness or swelling; (2) 1 point: Slight redness and swelling in a single area of the tarsal joint or ankle joint; (3) 2 points: Mild redness and swelling extending to the tarsal joint; (4) 3 points: Moderate redness and swelling in the ankle joint extending to the metatarsal bones; (5) 4 points: Severe redness and swelling in the ankle joint extending to the metatarsal bones, joint stiffness, and deformity.

RA-FLS extraction: Synovial tissue from CIA rats was taken, cut and enzymatically digested (Type II collagenase. Sigma-Aldrich, Shanghai, China). The incompletely digested tissue was filtered. Cells were placed in DMEM complete medium (Procell Life Technologies Ltd.) and incubated in an incubator at CO_2_.

Control group: without any drug treatment. LPS group: RA-FLS was treated with LPS (1 μg/ml) for 12 h. Methotrexate (MTX, 100 nM, Shanghai Mellin Biochemical Technology Co., Ltd.) group: add MTX and continue incubation for 12 h. WMP group: add WMP and continue incubation for 12 h.

### RA-FLS viability detection

Incubate RA-FLS (5 × 10^3^ cells/well) in a 96-well plate for 12 h. The WMP group was then exposed to serum concentrations containing drugs at 1%, 2%, 4%, 8%, 10%, 20%, 50%, and 100%. After an additional 24 h of incubation, 20 μl of CCK-8 was added to each well and incubated for 30 min. The cell viability was assessed by measuring the OD value of each group at 450 nm using an enzyme marker.

### RA-FLS migration detection

Inoculate RA-FLS (5 × 10^4^ cells/well) into a 6-well plate and incubate for 12 h, then use a 200-μl gun tip to scratch cells perpendicular to the well plate. Gently rinse off the floating cells from each well with PBS. Re-add the appropriate drug to each group. Each group was photographed and recorded at 0 h and 12 h, and the migration distance of RA-FLS in each group was calculated.

### RA-FLS invasion detection

The RA-FLS (1 × 10^4^cells/well) pretreated with LPS for 12 h was inoculated into materigel-coated transwell chambers, and the corresponding dose of drug was added and incubated for 12 h. After cell culture was completed, each group of transwells was sequentially fixed, stained, and rinsed with PBS. The number of cells attached to the outer ventricular membrane was observed under the microscope. Four fields of view were randomly selected for counting.

### Inflammatory factor testing

Cell supernatant preparation: Firstly, RA-FLS (1 × 105 cells/well) were seeded into a 6-well plate and incubated for 12 h. Next, all groups except for the control group were treated with the corresponding drugs and then incubated for 24 h. Finally, the supernatants from each group of cells were collected and placed into 1 ml centrifuge tubes, which were then centrifuged at 80×100*g* for 10 min at 4 °C. The supernatants were extracted and stored at − 80 °C for later use.

Preparation of rat serum: After administering the drug treatment, we obtained blood samples from each group of rats by collecting it from the eyeball vein. Blood at 4 °C, 80×100*g* centrifuge with force for 10 min, take the supernatant and store it at − 80 °C for later use.

ELISA kits are from Biyuntian Biotechnology Co. The specific test method is strictly according to the instruction of the kit.

### CIA rat modeling and grouping

The CIA rat model was replicated using the method outlined in “Effect of WMP on RA-FLS migration and invasion” section. The rats were allocated randomly into five groups (*n* = 5), namely the control, CIA, positive (methotrexate, MTX, 30 mg/kg), WMP sub-effective dose (0.5 g/kg) and WMP effective dose (2 g/kg) groups. After successful modeling, the control and model groups were orally administered physiological saline once a day for 15 consecutive days. In the positive group, methotrexate powder was dissolved in 0.1% carboxymethyl cellulose sodium solution at a dose of 30 mg/kg/day (MTX). WMP powder was dissolved in 0.1% carboxymethyl cellulose sodium solution and then orally administered at low and high doses of 0.5 g/kg/day and 2 g/kg/day respectively. The arthritis index was scored every 2 days for each group of rats. Fifteen days of treatment were carried out, followed by the collection of serum, synovial fluid, spleen, thymus, and knee tissue for analysis of pertinent physical and chemical indices.

### CIA rat immune organ index

Extract the thymus and spleen from each group of CIA rats, weigh them, and compare their weight to calculate the immune organ index of each rat group.

#### Histopathology assessment

Knee joints were extracted from the hind limbs of rats and subsequently decalcified using 10% ethylenediaminetetraacetic acid (EDTA). The joints were then fixed in paraffin, cut into 5 μm sections, and ultimately stained with hematoxylin–eosin and Anna Red O/Fast Green dyes, respectively.

#### Western blot

Cell sample processing: RA-FLS cells were seeded at a density of 5 × 10^5^ cells per well on a 6-well plate and incubated for 12 h. Replace the appropriate medication dosage and continue to cultivate for another 24 h. Subsequently, remove the culture medium and add 200 μl Radio immunoprecipitation assay (RIPA) cracking solution. Employ a centrifuge at 120×100*g* and 4 °C for 10 min. Lastly, extract the supernatant and store it at − 80 °C for future use. Synovial tissue processing: The synovium was lysed using 200 μl of RIPA lysate for every 20 mg of tissue. The tissue was homogenized in an ice bath employing a glass homogenizer until it completely dissolved.

The protein concentration of each group was determined using the Bicinchoninic Acid Assay (BCA) kit and standardized accordingly. Subsequently, the samples underwent electrophoresis and were transferred via electro transfer onto a PVDF membrane. Following a 2-h closure in 5% skimmed milk powder, each group was subjected to overnight incubation with the relevant primary antibody, washed thrice with Tris-buffered saline with Tween (TBST), and then incubated for 2 h with the secondary antibody. The PVDF membranes underwent development in a visualiser after being placed in Enhanced chemiluminescence (ECL) luminous reagent for 30 s. Technical term abbreviations were explained accordingly. The text adheres to a clear, concise, and formal writing style. The ImageJ software was used to analyze the gray scale values of each group of bands, and subsequent statistical analysis was conducted. The antibodies, including TLR4 (AY9008), TRAF6 (CY8163), NF-κB (CY5034), β-actin (AY0573) from Shanghai Abways Biotechnology Co., and IκB-α (bs-1287R), p-IκB-α (bs-2513R), MMP-2 (bs-20705R), MMP-9 (bs-7059R), TIMP-2 1 (bs-43009R), TIMP-12 (bs-0416R), Lamin B (bs-1840R) from Beijing Bioss Biotechnology Co. The reagents utilized in the experiment included BCA (P0010), TBST (ST673), ECL (P0018S), RIPA (P0013B) from Buffer Biyuntian Biotechnology Co.

All protein bands were detected by ImageJ 2.9.0 software for gray scale values and subsequent graphical analysis (Original images of all the western blot are in Additional file 1: supplemental material Figure 3).

#### Statistical analysis

All figures were performed using GraphPad Prism 6. Data were expressed as the mean ± standard deviation (SD). One-way analysis of variance (ANOVA) was used for statistical analysis of data, followed by *Tukey*’s post-hoc multiple comparison test. Statistical significance was defined as (#) or (*) *p* < 0.05, (##) or (**) *p* < 0.01, (###) or (***) *p* < 0.001.

### Supplementary Information

**Additional file 1.** WMP composition analysis and western blot of the original image.

## Data Availability

All data generated or analyzed during this study are included in this published article [and its supplementary information files].

## Abbreviations

WMP: Wu Mei Pill
RA: Rheumatoid arthritis
TLR: Toll-like receptor
LPS: Lipopolysaccharide
RIPA: Radio immunoprecipitation assay
ELISA: Enzyme-linked immunosorbent assay
TBST: Tris-buffered saline with Tween
UPLC-MS: Ultra-performance liquid chromatography/tandem mass spectrometry
CCK-8: Cell counting kit-8
TCM: Traditional Chinese medicine
DMARDs: Disease-modifying anti-rheumatic drugs
TNF-α: Tumor necrosis factor-α
IL-6: Interleukin-6
MyD88: Myeloid differentiation primary response protein 88
NLRP3: NOD-like receptor thermal protein domain associated protein 3
NF-κB: Nuclear factor kappa-B
IκB-α: Inhibitor κ B-α
MMP-2: Matrix metallopeptidase 2
MMP-9: Matrix metallopeptidase 9
TIMP-1: Tissue inhibitor of metalloproteinase 1
TIMP-2: Tissue inhibitor of metalloproteinase 2
MTX: Methotrexate

## References

[1] Radu A-F, Bungau SG (2021). Management of rheumatoid arthritis: an overview. Cells.

[2] Huang J, Fu X, Chen X, Li Z, Huang Y, Liang C (2021). Promising therapeutic targets for treatment of rheumatoid arthritis. Front Immunol.

[3] Chen J-Q, Szodoray P, Zeher M (2016). Toll-like receptor pathways in autoimmune diseases. Clin Rev Allergy Immunol.

[4] Fitzgerald KA, Kagan JC (2020). Toll-like receptors and the control of immunity. Cell.

[5] Park BS, Song DH, Kim HM, Choi B-S, Lee H, Lee J-O (2009). The structural basis of lipopolysaccharide recognition by the TLR4–MD-2 complex. Nature.

[6] Lu Y-C, Yeh W-C, Ohashi PS (2008). LPS/TLR4 signal transduction pathway. Cytokine.

[7] Ilchovska D, Barrow DM (2021). An overview of the NF-kB mechanism of pathophysiology in rheumatoid arthritis, investigation of the NF-kB ligand RANKL and related nutritional interventions. Autoimmun Rev.

[8] Yoo HJ, Byun H-J, Kim B-R, Lee KH, Park S-Y, Rho SB (2012). DAPk1 inhibits NF-κB activation through TNF-α and INF-γ-induced apoptosis. Cell Signal.

[9] Piccinini AM, Williams L, McCann FE, Midwood KS (2016). Investigating the role of toll-like receptors in models of arthritis. Methods Mol Biol.

[10] Frisenda S, Perricone C, Valesini G (2013). Cartilage as a target of autoimmunity: a thin layer. Autoimmun Rev.

[11] Bartok B, Firestein GS (2010). Fibroblast-like synoviocytes: key effector cells in rheumatoid arthritis. Immunol Rev.

[12] Filer A (2013). The fibroblast as a therapeutic target in rheumatoid arthritis. Curr Opin Pharmacol.

[13] Tanaka T, Narazaki M, Kishimoto T (2018). Interleukin (IL-6) immunotherapy. Cold Spring Harb Perspect Biol.

[14] Zavvar M, Assadiasl S, Zargaran S, Akhtari M, Poopak B, Dinarvand R (2020). Adoptive treg cell-based immunotherapy: frontier therapeutic aspects in rheumatoid arthritis. Immunotherapy.

[15] Jakobsson P, Robertson L, Welzel J, Zhang M, Zhihua Y, Kaixin G (2022). Where traditional Chinese medicine meets Western medicine in the prevention of rheumatoid arthritis. J Intern Med.

[16] Wang J, Ding K, Wang Y, Yan T, Xu Y, Deng Z (2022). Wumei pill ameliorates AOM/DSS-induced colitis-associated colon cancer through inhibition of inflammation and oxidative stress by regulating S-adenosylhomocysteine hydrolase- (AHCY-) mediated hedgehog signaling in mice. Oxid Med Cell Longev.

[17] Ding X, Sun X, Wang Z, Zheng Q, Yu X, Hao W (2018). The effects of Wumei pill on TLRs/NF-kB signaling pathway in rats with diarrhea-predominant irritable bowel syndrome. Pak J Zool.

[18] Lu D-X, Liu F, Wu H, Liu H-X, Chen B-Y, Yan J (2022). Wumei pills attenuates 5-fluorouracil-induced intestinal mucositis through Toll-like receptor 4/myeloid differentiation factor 88/nuclear factor-κB pathway and microbiota regulation. World J Gastroenterol.

[19] Yan S, Wang P, Wei H, Jia R, Zhen M, Li Q (2022). Treatment of ulcerative colitis with Wu-Mei-Wan by inhibiting intestinal inflammatory response and repairing damaged intestinal mucosa. Phytomedicine.

[20] Yu M-H, Hung T-W, Wang C-C, Wu S-W, Yang T-W, Yang C-Y (2021). Neochlorogenic acid attenuates hepatic lipid accumulation and inflammation via regulating miR-34a in vitro. Int J Mol Sci.

[21] Wu J, Luo Y, Jiang Q, Li S, Huang W, Xiang L (2019). Coptisine from Coptis chinensis blocks NLRP3 inflammasome activation by inhibiting caspase-1. Pharmacol Res.

[22] Yang D, Tan Y, Xie X, Xiao W, Kang J (2023). Zingerone attenuates Ti particle-induced inflammatory osteolysis by suppressing the NF-κB signaling pathway in osteoclasts. Int Immunopharmacol.

[23] Aletaha D, Smolen JS (2018). Diagnosis and management of rheumatoid arthritis. JAMA.

[24] Medzhitov R, Preston-Hurlburt P, Janeway CA (1997). A human homologue of the Drosophila Toll protein signals activation of adaptive immunity. Nature.

[25] Li J, Jia Q, Liu Y, Chen D, Fang Z, Liu Y (2022). Different structures of arabinoxylan hydrolysates alleviated Caco-2 cell barrier damage by regulating the TLRs/MyD88/NF-κB pathway. Foods.

[26] Takeda K, Akira S (2004). TLR signaling pathways. Semin Immunol.

[27] Xiao L, Zhong M, Huang Y, Zhu J, Tang W, Li D (2020). Puerarin alleviates osteoporosis in the ovariectomy-induced mice by suppressing osteoclastogenesis via inhibition of TRAF6/ROS-dependent MAPK/NF-κB signaling pathways. Aging (Albany NY).

[28] Elshabrawy HA, Essani AE, Szekanecz Z, Fox DA, Shahrara S (2017). TLRs, future potential therapeutic targets for RA. Autoimmun Rev.

[29] Kiener HP, Watts GFM, Cui Y, Wright J, Thornhill TS, Sköld M (2010). Synovial fibroblasts self-direct multicellular lining architecture and synthetic function in three-dimensional organ culture. Arthritis Rheum.

[30] Itoh Y (2017). Metalloproteinases in rheumatoid arthritis: potential therapeutic targets to improve current therapies. Prog Mol Biol Transl Sci.

[31] Bormann T, Maus R, Stolper J, Tort Tarrés M, Brandenberger C, Wedekind D (2022). Role of matrix metalloprotease-2 and MMP-9 in experimental lung fibrosis in mice. Respir Res.

[32] Jiang Y, Sang W, Wang C, Lu H, Zhang T, Wang Z (2018). Oxymatrine exerts protective effects on osteoarthritis via modulating chondrocyte homoeostasis and suppressing osteoclastogenesis. J Cell Mol Med.

[33] Kim KS, Choi HM, Lee Y-A, Choi IA, Lee S-H, Hong S-J (2011). Expression levels and association of gelatinases MMP-2 and MMP-9 and collagenases MMP-1 and MMP-13 with VEGF in synovial fluid of patients with arthritis. Rheumatol Int.

[34] Bourboulia D, Stetler-Stevenson WG (2010). Matrix metalloproteinases (MMPs) and tissue inhibitors of metalloproteinases (TIMPs): positive and negative regulators in tumor cell adhesion. Semin Cancer Biol.

[35] Khokha R, Murthy A, Weiss A (2013). Metalloproteinases and their natural inhibitors in inflammation and immunity. Nat Rev Immunol.

[36] Kim KS, Kim M-H, Yeom M, Choi HM, Yang H-I, Yoo MC (2012). Arthritic disease is more severe in older rats in a kaolin/carrageenan-induced arthritis model. Rheumatol Int.

[37] Bevaart L, Vervoordeldonk MJ, Tak PP (2010). Collagen-induced arthritis in mice.

