# Supplementary material for: Study on the mechanism of action of Wu Mei Pill in inhibiting rheumatoid arthritis through TLR4-NF-κB pathway
Source: J Orthop Surg Res. 2024 Jan 13;19:65. doi: 10.1186/s13018-024-04551-z (PMC10787417; doi:10.1186/s13018-024-04551-z)

Supplementary Table 1

TCM Positive Ion Identification

No.	Compound	m/z	Reten	Adduct	Formula	Score	Fragme	Mass E	Fragment	Peak Area	Ingredient_id	Source
1	0.96_192.0269	210.0607	0.96	M+H-2	C6H8O7	46.80	64.30	-0.73	68.9984, 1	##########	Citric acid	wumeirou
2	3.81_268.1037	268.1037	3.81	M+H	C10H13N5	44.40	26.00	-1.20	136.0618,	5948240.96	Adenine nucleoside	danggui,ganjiang
3	7.38_162.0315	163.0387	7.38	M+H-H	C9H6O3	52.10	62.90	-1.49	89.0395, 1	##########	Umbelliferone	danggui
4	9.38_356.1484	356.1484	9.38	M+NH4	C20H18O5	41.70	14.80	-2.39	58.0666, 3	##########	Glepidotin a	gancao
5	9.86_358.1640	358.1640	9.86	M+NH4	C20H20O5	40.80	10.30	-2.59	58.0666, 3	4406196.32	2,4,2',4'-tetrahydroxy	gancao
6	10.05_354.094	355.1015	####	M+H-H	C16H18O9	45.00	32.20	-2.56	70.0664, 3	##########	Neochlorogenic acid	wumeirou
7	10.14_177.054	177.0544	####	M+H-H	C10H10O4	50.30	54.30	-1.10	117.0339,	##########	Vanillin acetate	danggui
8	10.54_358.164	358.1640	####	M+NH4	C20H20O5	41.90	16.40	-2.50	58.0666, 3	4510828.35	Licocoumarone	gancao
9	11.10_312.122	312.1225	####	M+NH4	C18H14O4	48.70	48.60	-1.82	312.1228,	7010557.57	7-acetoxy-2-methyli	gancao
10	11.54_358.200	358.2005	####	M+NH4	C21H24O4	39.70	5.22	-2.25	58.0666, 3	5430554.33	Gancaonin z	gancao
11	11.58_480.162	481.1701	####	M+H, M	C23H28O1	47.50	46.70	-0.75	105.0342,	8760825.17	Albiflorin	baishao
12	11.77_340.153	340.1539	####	M+NH4	C20H18O4	50.80	58.90	-1.28	265.0863,	9236301.90	Kanzonol b	gancao
13	12.34_356.185	356.1850	####	M+NH4	C21H22O4	51.40	62.50	-1.89	58.0666, 3	##########	Gancaonin x	gancao
14	12.86_340.153	340.1538	####	M+NH4	C20H18O4	47.10	41.30	-1.69	309.099, 3	4033255.96	Shinpterocarpin	gancao
15	12.99_356.184	356.1849	####	M+NH4	C21H22O4	46.40	37.90	-2.08	206.1176,	5202208.54	4'-o-methylglabridin	gancao,ganjiang
16	13.09_338.101	338.1015	####	M+H	C19H15NO	49.60	55.60	-2.24	338.1018,	##########	9-ethoxyaristololacta	ganjiang
17	13.11_177.054	177.0544	####	M+H-H	C10H10O4	52.10	62.90	-1.24	117.0339,	##########	4-hydroxy-3-methox	danggui
18	13.30_340.153	340.1538	####	M+NH4	C20H18O4	48.80	49.20	-1.66	309.0992,	8430769.24	Glabrene	gancao
19	13.89_356.184	356.1848	####	M+H	C21H25NO	47.20	42.00	-2.34	192.1019,	##########	Tetrahydropalmatine	gancao
20	14.60_177.054	177.0543	####	M+H-H	C10H10O4	52.30	95.40	-1.37	117.034, 8	2357221.98	Ferulic acid	danggui
21	14.75_354.169	354.1692	####	M+H	C21H23NO	47.30	42.00	-2.11	323.1149,	##########	Delta-cadinene	ganjiang
22	14.83_368.148	368.1485	####	M+NH4	C21H18O5	50.00	56.20	-2.03	368.1494,	6560287.12	Glycyrrhizol b	gancao
23	14.85_338.138	338.1380	####	M+	C20H20NO	49.20	53.60	-3.78	307.0836,	4486542.50	Jatrorrhizine	gancao
24	15.11_351.106	351.1065	####	M+Na	C15H20O8	49.60	55.60	4.46	147.0441,	6357502.23	Paeonoside	baishao
25	15.19_320.090	320.0908	####	M+	C19H14NO	40.90	96.00	-4.73	320.0917,	##########	Coptisine	huanglian
26	15.27_257.080	257.0802	####	M+H	C15H12O4	51.70	63.30	-2.37	137.0234,	##########	Liquiritigenin	gancao
27	15.33_147.043	147.0438	####	M+H	C9H6O2	51.50	61.20	-2.06	147.0441,	5936461.07	Coumarin	guizhi
28	15.63_336.122	336.1221	####	M+	C20H18NO	33.40	55.80	-4.54	322.1067,	##########	Epiberberine	huanglian
29	17.33_418.125	419.1329	####	M+H-2	C21H22O9	50.80	61.40	-1.80	69.0347, 1	##########	Neoisoliquiritin	gancao
30	17.33_273.075	273.0752	####	M+H	C15H12O5	49.40	52.00	-1.99	153.0182,	##########	Naringenin	gancao

31	17.49_354.159	354.1593	####	M+NH	C20H18Cl	51.00	93.50	5.98	353.1573,	##########	Berberine hydrochlo	huanglian,huangbai
32	17.62_207.101	207.1014	####	M+H-H	C12H16O	48.80	89.50	-0.88	207.1017,	2009774.52	Senkyunolide i	danggui
33	18.32_303.085	303.0857	####	M+H	C16H14O	44.70	28.10	-1.90	153.0182,	##########	3,4,3',4'-tetrahydrox	gancao
34	18.44_604.311	604.3110	####	M+NH	C32H42O	55.60	80.20	-1.10	604.3112,	##########	Yuanhuadine	gancao
35	19.03_132.057	133.0647	####	M+H-H	C9H8O	48.30	42.90	-0.67	115.0547,	##########	cinnamaldehyde	guizhi
36	19.13_574.300	574.3005	####	M+H	C31H43N	57.10	88.50	-1.09	574.301, 1	##########	Benzoylhypacoitine	heishunpian
37	19.86_290.138	290.1380	####	M+H-H	C16H21N	47.40	42.60	-2.17	164.0705,	4469101.72	Foliosidine	danggui
38	20.50_246.075	246.0755	####	M+NH	C13H8O4	39.00	0.00	-2.63	231.0526,	##########	Farnesol	ganjiang
39	20.81_632.305	632.3054	####	M+H	C33H45N	47.70	87.20	-1.78	105.0342,	352986.68	Mesaconitine	heishunpian
40	21.05_302.137	302.1379	####	M+H	C17H19N	41.60	13.90	-2.56	242.1174,	4060589.59	Crinamine	danggui
41	21.39_287.090	287.0905	####	M+H	C16H14O	39.00	1.40	-3.08	153.0182,	##########	Licochalcone b	gancao
42	21.54_616.310	616.3105	####	M+H	C33H45N	51.20	96.80	-1.75	105.0342,	1860826.98	Hypaconitine	heishunpian
43	21.64_162.067	163.0749	####	M+H-H	C10H10O	47.50	41.50	-2.70	115.0547,	##########	Methylglyoxal	ganjiang
44	21.81_646.320	646.3209	####	M+H	C34H47N	48.20	91.30	-1.93	105.0342,	276642.31	Aconitine	heishunpian
45	22.53_216.041	217.0491	####	M+H, M	C12H8O4	48.00	44.00	-2.10	91.0549, 1	##########	Maxacalcitol	ganjiang
46	22.98_358.144	376.1779	####	M+H-H	C17H26O	49.40	58.90	-2.67	137.0599,	4269480.23	6-gingesulfonic acid	ganjiang
47	23.94_195.065	195.0650	####	M+H	C10H10O	50.00	80.30	-1.08	57.0349, 1	2175477.05	Kakuol	xixin
48	25.30_192.114	193.1220	####	M+H-2	C12H16O	46.50	35.30	-1.70	112.0762,	4966864.30	Senkyunolide	danggui
49	25.60_372.119	373.1272	####	M+H, M	C20H20O	41.50	14.20	-2.56	395.11, 36	8657137.18	Tangeretin	zhike
50	26.15_210.161	228.1953	####	M+H-2	C13H22O	43.40	21.60	-2.19	95.0864, 6	4189141.70	N-hexadecanoic acid	ganjiang
51	26.20_356.161	357.1689	####	M+H, M	C21H24O	41.00	11.40	-2.21	379.1531,	4562226.92	Gingerenone b	ganjiang
52	26.32_426.203	427.2108	####	M+H-2	C25H30O	44.20	28.40	-1.62	95.0136, 4	4367976.93	Rhinacanthin i	ganjiang
53	26.46_294.182	277.1790	####	M+H-2	C17H26O	51.40	62.70	-2.76	259.1692,	##########	Gingerone, methyl e	ganjiang
54	26.67_344.161	327.1582	####	M+H-H	C20H24O	39.10	5.95	-2.60	193.0496,	4060231.94	5r-hydroxy-1-(4-hyd	ganjiang
55	26.85_440.364	423.3610	####	M+H-2	C30H48O	39.80	7.04	-2.51	404.1423,	5273744.06	Sebiferic acid	danggui
56	27.08_353.137	353.1373	####	M+H	C21H20O	49.00	52.70	-2.93	353.138, 6	5003290.84	Gancaonin a	gancao
57	27.22_486.333	469.3305	####	M+H-H	C30H46O	52.80	67.40	-1.60	119.086, 1	##########	Licorice-saponin g2	gancao
58	27.30_490.219	508.2534	####	M+H, M	C26H34O	50.90	58.40	-1.54	137.0597,	9425242.44	3,5-diacetoxy-1-(4-h	ganjiang
59	27.51_460.208	478.2427	####	M+NH	C25H32O	47.50	41.80	-1.85	137.0598,	4021863.57	(3s,5s)-3,5-diacetox	ganjiang
60	27.63_190.098	191.1062	####	M+H-2	C12H14O	51.00	96.40	-2.74	155.0844,	##########	Ligustilide	danggui
61	27.94_188.083	189.0907	####	M+H-2	C12H12O	46.30	34.70	-1.73	153.0699,	4257585.19	(z)-3-butylidenephth	danggui
62	28.12_402.130	403.1379	####	M+H, M	C21H22O	41.40	13.10	-2.07	425.1198,	##########	Nobiletin	zhike

63	28.45_308.198	291.1948	####	M+H-2	C18H28O4	49.90	55.20	-2.28	98.9849, 1	5135808.83	2-methoxy-4-[2-[(4r	ganjiang
64	28.69_432.141	433.1486	####	M+H, M	C22H24O9	42.40	16.20	-1.62	425.0845,	##########	(-)-medicocarpin	gancao
65	28.96_336.099	337.1063	####	M+H-H	C20H16O5	44.00	26.00	-2.27	137.0598,	5275252.16	Kanzonol w	gancao
66	29.54_354.109	337.1062	####	M+H-2	C20H18O6	52.10	81.00	-2.17	203.0855,	##########	Asarinin	xixin
67	29.72_439.356	439.3565	####	M+H-H	C30H48O3	50.00	56.60	-1.25	95.0864, 1	5737265.78	3-epioleanolic acid	baishao,gancao
68	29.96_470.338	453.3355	####	M+H-H	C30H46O4	51.40	64.80	-1.72	471.3473,	##########	3beta,23-dihydroxyc	baishao
69	30.04_276.171	277.1792	####	M+H, M	C17H24O3	53.90	74.70	-2.31	299.1618,	##########	6-shogaol	ganjiang
70	30.08_312.135	313.1426	####	M+H-H	C19H20O4	42.80	20.50	-2.81	159.044, 2	9722669.98	Gancaonin v	gancao
71	30.20_470.338	453.3355	####	M+H-H	C30H46O4	52.90	68.70	-1.67	471.3468,	##########	3β-hydroxy-11-oxoo	baishao
72	30.27_322.213	305.2104	####	M+H-2	C19H30O4	46.50	37.90	-2.27	287.2003,	##########	(8)-gingerol	ganjiang
73	30.39_326.151	327.1584	####	M+H, M	C20H22O4	40.10	6.32	-2.16	178.0261,	6477470.28	Gingerglycolipid a	ganjiang
74	30.39_386.172	409.1614	####	M+NH4	C22H26O6	38.80	0.18	-1.98	193.0497,	6628542.17	Gingerenone-c	ganjiang
75	30.45_1108.60	########	####	M+H-H	C54H92O2	56.20	88.40	-1.70	85.0294, 9	7907296.07	Ginsenoside rb1	renshen
76	30.74_470.338	453.3355	####	M+H-H	C30H46O4	52.70	67.30	-1.74	471.3474,	##########	18alpha-glycyrrhetir	gancao
77	31.27_1078.59	########	####	M+H-H	C53H90O2	55.00	96.20	-2.07	1101.5822	3230984.75	Ginsenoside rb2	renshen
78	31.51_229.085	229.0853	####	M+H	C14H12O3	40.10	5.30	-2.53	229.0859,	9799356.96	Resveratrol	baishao,danggui
79	31.66_471.346	471.3460	####	M+H	C30H46O4	51.80	64.80	-1.87	119.0859,	4401327.10	Apioglycyrrhizin de	gancao
80	31.84_380.218	398.2525	####	M+NH4	C21H32O6	43.40	24.40	-3.14	403.2101,	##########	Dicyclopropyl keton	ganjiang
81	31.84_380.197	381.2051	####	M+H, M	C24H28O4	46.80	41.30	-2.43	403.188, 2	##########	Levistolid a	danggui
82	31.84_380.198	398.2322	####	M+H-H	C24H28O4	47.30	41.60	-0.89	137.0595,	7148389.82	6,7,3',8'-diligustilide	danggui
83	32.22_380.197	381.2051	####	M+H, M	C24H28O4	46.30	38.70	-2.45	213.0886,	##########	Angelicide	danggui
84	32.22_191.106	191.1062	####	M+H-H	C12H16O3	46.50	37.00	-1.97	191.1066,	5184481.44	[1]-paradol	ganjiang
85	32.50_191.106	191.1063	####	M+H	C12H14O2	45.90	33.90	-2.12	191.1065,	4728584.70	Butylphthalide	danggui
86	32.58_278.151	301.1403	####	M+H, M	C16H22O4	39.40	2.21	-2.64	301.1402,	5771494.33	Diisobutyl phthalate	gancao
87	32.60_330.335	330.3358	####	M+NH4	C20H40O2	47.10	41.70	-2.82	330.3365,	6046423.20	Arachidic acid	gancao,ganjiang
88	33.21_194.093	177.0907	####	M+H-H	C11H14O3	50.20	54.50	-1.81	117.0703,	9420378.04	Zingiberene	ganjiang
89	33.21_350.244	333.2414	####	M+H-2	C21H34O4	47.20	42.20	-2.85	315.2314,	##########	(10)-gingerol	ganjiang
90	33.23_310.215	310.2157	####	M+	C18H30O4	38.10	0.12	4.25	184.0756,	4783607.38	6-methylgingediol	ganjiang
91	33.25_137.059	137.0595	####	M+H	C8H8O2	40.50	5.11	-1.82	137.0598,	5880460.44	Phenylacetic acid	danggui
92	33.27_304.203	305.2103	####	M+H, M	C19H28O3	45.00	30.50	-2.58	98.9849, 3	4657764.32	(e)-1-(4-hydroxy-3-r	ganjiang
93	33.31_193.049	193.0492	####	M+H	C10H8O4	53.80	72.30	-1.82	178.0259,	##########	Chrysatropic acid	danggui,gancao
94	33.31_394.234	412.2684	####	M+NH4	C22H34O6	47.90	46.60	-2.37	151.0754,	9054539.20	6-methylgingediacet	ganjiang

95	33.33_380.197	381.2051	####	M+H, M	C24H28O4	44.30	29.30	-2.43	403.1884,	4944701.70	Levistilide a	danggui
96	34.78_297.241	297.2417	####	M+H	C18H32O3	48.20	46.90	-2.58	81.0709, 1	4547347.56	Rhizobialide	gancao
97	35.53_378.275	361.2725	####	M+H-2	C23H38O4	44.30	29.10	-3.20	343.2626,	5184491.32	[12]-gingerol	ganjiang
98	35.74_332.233	333.2412	####	M+H, M	C21H32O3	43.30	24.00	-3.65	355.2242,	##########	[10]-shogaol	ganjiang
99	36.57_348.228	331.2256	####	M+H-H	C21H32O4	46.60	40.50	-3.35	91.0585, 1	##########	10-gingerdione	ganjiang
100	36.63_262.228	280.2625	####	M+H-H	C18H30O	45.10	31.90	-3.76	91.055, 11	7858804.02	Farnesylacetone	danggui
101	36.65_454.343	455.3507	####	M+H-H	C30H46O3	46.40	40.70	-2.84	95.0863, 1	4537625.41	Licoricesaponin c2 c	gancao
102	37.20_354.275	377.2649	####	M+H-H	C21H38O4	42.10	18.40	-3.79	67.0555, 8	##########	1-monolinolein	ganjiang
103	38.10_456.358	439.3556	####	M+H-H	C30H48O3	47.30	43.60	-3.10	95.0864, 2	##########	3-epikatonic acid	gancao
104	38.32_376.260	359.2567	####	M+H-H	C23H36O4	43.40	25.30	-3.51	145.0284,	4851973.13	[12]-gingerdione	ganjiang
105	38.41_430.307	431.3144	####	M+H, M	C27H42O4	46.10	38.50	-2.79	137.0596,	4479877.86	[6]-gingerdiol (2e)-g	ganjiang
106	39.85_390.275	413.2648	####	M+H, M	C24H38O4	40.30	9.94	-3.66	413.2656,	7216326.76	Acetoxy-[10]-ginger	ganjiang
107	41.42_297.147	297.1473	####	M+Na	C17H22O3	45.00	33.80	4.37	163.0389,	4650158.65	2-butyl-5-[2-(4-hydr	ganjiang
108	42.96_758.567	758.5676	####	M+	C42H81NO	40.80	8.82	-3.19	86.0974, 1	9631912.49	Lecithin	danggui

TCM negative ion identification

No.	Compound	m/z	Retentic	Adducts	Formula	Score	Fragme	Mass Err	Fragments	Peak Area	Ingredient_id	Source
109	6.35_705.1674m/	705.1674	6.35	M+FA-H	C31H32O16	40.50	11.60	0.29	111.0063, 339.0495,	4580093	3,4-di-o-caffeoyl-5-(	gancao
110	9.03_289.0715m/	289.0715	9.03	M-H	C15H14O6	56.70	86.10	-0.74	289.072, 80.9624, 10	1088442	Catechin	baishao
111	9.93_495.1504m/	495.1504	9.93	M-H	C23H28O12	50.40	65.70	-0.79	137.022, 93.0319, 49	284137	Oxypaeoniflorin	baishao
112	10.57_354.0948n	353.0875	10.57	M-H, M+Na-2H	C16H18O9	39.50	2.51	-0.88	135.0428, 173.0436,	########	Scopolin	danggui
113	11.56_480.1627n	525.1609	11.56	M-H, M+FA-H	C23H28O11	47.00	39.30	-0.88	111.0062, 121.027, 1	########	Albiflorin r1	baishao
114	11.73_289.0715n	289.0715	11.73	M-H	C15H14O6	44.20	24.90	-0.85	289.0704, 109.0268,	2489530	Cianidanol	baishao
115	12.98_449.1450n	449.1450	12.98	M+FA-H	C21H24O8	42.30	13.70	-0.82	121.027, 189.0176, 3	7987769	Angeliferulate	danggui
116	12.98_480.1626n	525.1608	12.98	M-H, M+FA-H,	C23H28O11	50.20	86.80	-1.35	121.027, 449.1453	########	Paeoniflorin	baishao
117	13.43_335.0771n	335.0771	13.43	2M-H	C8H8O4	43.40	25.20	-0.33	161.0224, 133.0271,	3182243	Verbenol	danggui,gan
118	13.46_593.1508n	593.1508	13.46	M-H	C27H30O15	47.80	43.20	-0.65	593.1488, 297.0771,	5170978	Vicenin 2	gancao
119	15.00_595.1667n	595.1667	15.00	M+FA-H	C26H30O13	41.80	11.50	-0.22	151.0014, 135.0428,	2318808	Liquiritin apioside	gancao
120	15.22_418.1261n	417.1189	15.22	M-H, 2M-H	C21H22O9	53.20	80.30	-0.63	119.0481, 255.0658,	########	Liquiritin	zhigancao
121	15.49_550.1685n	549.1612	15.49	M-H, 2M-H	C26H30O13	49.20	46.70	-0.28	119.0478, 255.0656,	########	Isoliquiritin apioside	gancao
122	17.24_579.1713n	579.1713	17.24	M-H	C27H32O14	48.30	43.70	-1.14	151.0013, 119.0478,	########	Naringin	zhike,gancao
123	17.42_609.1455n	609.1455	17.42	M-H	C27H30O16	56.20	83.40	-1.07	271.0247, 300.0272,	4037971	Neoisorutin	gancao
124	18.22_610.1891n	609.1818	18.22	M-H, M+Na-2H	C28H34O15	47.60	48.60	-1.11	609.1822, 301.0713,	########	Neohesperidin	zhike
125	18.36_623.1613n	623.1613	18.36	M-H	C28H32O16	44.90	28.30	-0.72	315.0508, 300.0273,	2291936	Narcissoside	gancao
126	18.87_578.1631n	577.1558	18.87	M-H, 2M-H	C27H30O14	39.70	3.14	-0.75	269.0451, 577.1569,	4237207	Isoviolanthin	gancao
127	19.03_607.1668n	607.1668	19.03	M+FA-H	C27H30O13	41.30	13.20	-0.15	284.0323, 299.0557,	########	Glycyroside	gancao
128	19.61_549.1612n	549.1612	19.61	M-H	C26H30O13	48.30	43.60	-0.31	119.0478, 255.0658,	3946928	Licuraside	gancao
129	19.81_417.1187n	417.1187	19.81	M-H	C21H22O9	49.40	51.90	-0.87	119.0478, 255.0657,	4195141	Neoliquiritin	gancao
130	22.75_584.1890n	629.1872	22.75	M-H, M+FA-H	C30H32O12	48.70	70.40	-0.64	121.0271, 553.1746,	6707143	Benzoylpaeoniflorin	baishao
131	22.86_357.1374n	357.1374	22.86	M-H	C17H26O6S	47.90	42.50	-0.81	80.9625, 357.1382, 1	########	6-gingesulfonic acid	ganjiang
132	23.31_946.5501n	991.5483	23.31	M-H, M+FA-H	C48H82O18	47.70	82.80	-0.02	945.5433, 71.0111, 9	########	Ginsenoside re	renshen
133	23.47_835.4626n	835.4626	23.47	2M-H	C24H34O6	32.70	0.00	-1.48	835.4507, 111.006, 9	3113298	Phyllanthin	danggui
134	23.47_800.4920n	845.4902	23.47	M-H, M+FA-H	C42H72O14	39.20	32.00	-0.22	845.4913, 59.0112, 7	########	Ginsenoside rg1	renshen
135	23.79_432.1778n	431.1705	23.79	M-H, M+Na-2H	C23H28O8	48.50	45.60	-1.49	453.1531, 122.035, 1	########	3-acetoxy-1,5-epoxy	ganjiang
136	24.56_1224.5759	1223.5686	24.56	M-H, M+FA-H	C57H92O28	52.10	89.30	-1.33	1223.5707, 681.3856	########	Platycodin d	jiegeng
137	26.49_355.1183n	355.1183	26.49	M-H	C20H20O6	49.10	50.20	-1.21	125.022, 355.1159, 5	5348009	Sigmoidin b	gancao
138	26.76_984.4558n	983.4486	26.76	M-H, M+Na-2H	C48H72O21	55.50	79.20	-0.79	843.3787, 1005.4344	########	Licoricesaponin a3	gancao
139	27.08_503.3374n	503.3374	27.08	M+FA-H	C29H46O4	54.80	77.10	-0.93	503.3378, 111.0062,	2153936	24-hydroxy-11-deox	gancao
140	27.41_356.1620n	355.1547	27.41	M-H, M+FA-H,	C21H24O5	47.40	42.30	-1.05	355.1547, 135.0428,	9382649	Glyasperin c	gancao
141	27.93_293.1756n	293.1756	27.93	M-H	C17H26O4	43.90	92.00	-0.81	80.9625, 221.1534, 2	692994	6-gingerol	ganjiang
142	28.02_354.1463n	353.1390	28.02	M-H, 2M-H	C21H22O5	49.70	53.20	-1.22	353.1388, 637.6212,	7362540	Dehydroglyasperin (	gancao
143	28.24_353.1025n	353.1025	28.24	M-H	C20H18O6	49.00	50.30	-1.62	125.022, 353.1028, 5	########	Glyasperin f	gancao

144	28.26_351.0873n	351.0873	28.26	M-H	C20H16O6	50.00	54.00	-0.32	125.0219, 57.032, 35	3119795	Semilicoisoflavone	gancao
145	28.78_367.1180n	367.1180	28.78	M-H	C21H20O6	47.20	41.70	-1.88	367.1195, 309.0406,	9092789	Gancaonin b	gancao
146	28.92_838.3986n	837.3913	28.92	M-H, M+Na-2H	C42H62O17	44.80	25.80	-0.15	859.3724, 72.9903, 7	########	Licoricesaponine g2	gancao
147	29.32_354.1100n	353.1027	29.32	M-H, 2M-H	C20H18O6	47.70	43.60	-0.95	353.1031, 297.0401,	4332350	Licoflavonol	gancao
148	29.46_838.3986n	837.3914	29.46	M-H, M+Na-2H	C42H62O17	43.20	16.90	-0.07	859.374, 115.9177, 7	########	Licorice-saponin g2	gancao
149	29.84_843.3767n	843.3767	29.84	M+Na-2H	C42H62O16	55.60	84.10	-2.11	843.379, 71.0112, 72	3113385	Glyyunnanprosapog	gancao
150	29.88_821.3963n	821.3963	29.88	M-H	C53H90O22	43.10	34.10	-0.27	821.3975, 351.0564,	########	Glycyrrhizic acid	zhigancao
151	30.43_352.0942n	351.0869	30.43	M-H, 2M-H	C20H16O6	43.70	23.90	-1.53	351.0876, 125.0221,	8678937	Licoisoflavone b	gancao
152	30.56_1078.5917	1123.5899	30.57	M-H, M+Na-2H	C53H90O22	37.70	71.30	-0.55	1077.585, 89.0217, 1	########	Ginsenoside rc	renshen
153	30.68_822.4039n	821.3966	30.68	M-H, M+Na-2H	C42H62O16	46.20	31.60	0.16	843.377, 113.0211, 7	########	Licorice-saponin k2	gancao
154	31.22_353.1026n	353.1026	31.22	M+FA-H	C19H16O4	51.30	61.80	-1.43	353.1028, 241.05, 26	4009154	Glabrocoumarone a	gancao
155	31.78_370.1774n	369.1701	31.78	M-H, M+FA-H,	C22H26O5	43.50	23.20	-1.73	369.1701, 135.0428,	########	Glyasperin d	gancao
156	31.85_707.2859n	707.2859	31.85	2M-H	C21H22O5	50.70	73.40	-0.40	707.2827, 499.176, 3	3741360	Licochalcone g	gancao
157	32.14_824.4198n	823.4125	32.14	M-H, M+Na-2H	C42H64O16	45.60	31.10	0.40	845.3948, 113.0219,	2817009	Licorice-saponin j2	gancao
158	32.16_289.1443n	289.1443	32.16	M-H	C17H22O4	51.50	61.10	-0.79	134.035, 149.0585, 1	2217120	[6]-dehydrogingerdi	ganjiang
159	32.23_946.5501n	991.5483	32.23	M-H, M+FA-H	C48H82O18	50.30	70.60	0.03	945.5428, 71.0111, 8	########	Ginsenoside rd	renshen
160	32.39_486.3340n	485.3267	32.39	M-H, M+FA-H	C30H46O5	54.80	80.50	-1.10	99.9226, 116.9258, 4	8312237	18alpha-hydroxygly	gancao
161	32.43_423.1810n	423.1810	32.43	M-H	C25H28O6	50.70	58.70	-0.73	193.0854, 229.0862,	########	3'(γ,γ-dimethylallyl)	gancao
162	32.54_824.4198n	823.4125	32.54	M-H, M+Na-2H	C42H64O16	46.90	37.00	0.42	845.3963, 115.9181,	9636392	Licoricesaponine j2	gancao
163	33.04_808.4248n	807.4175	33.04	M-H, M+Na-2H	C42H64O15	49.40	48.50	0.35	829.3995, 71.0112, 7	3575335	Licoricesaponin b2	gancao
164	33.22_421.1656n	421.1656	33.22	M-H	C25H26O6	47.90	44.00	-0.25	421.1651, 352.0952,	3459652	Glyasperin a	gancao
165	33.62_421.1653n	421.1653	33.62	M-H	C25H26O6	49.90	54.60	-0.77	193.0854, 421.1635,	4108928	Glyurallin b	gancao
166	33.69_472.3544n	471.3471	33.69	M-H, M+FA-H,	C30H48O4	51.00	58.90	-1.85	471.3476, 453.3373	6645909	Licoricesaponin j2 c	gancao
167	33.87_421.1652n	421.1652	33.87	M-H	C25H26O6	47.00	40.50	-1.14	421.1654, 309.0386,	########	Isoangustone a	gancao
168	34.14_676.3669n	721.3651	34.14	M-H, M+FA-H	C33H56O14	48.80	45.00	-0.12	675.3607, 277.2171,	4967148	Gingerglycolipid b	ganjiang
169	34.54_405.1703n	405.1703	34.54	M-H	C25H26O5	44.50	27.80	-1.17	405.1711, 307.0615	3062176	Kanzonol z	gancao
170	34.70_471.3473n	471.3473	34.70	M-H	C30H48O4	52.20	63.80	-1.45	471.3474	2124406	Anemosapogenin	baishao
171	35.28_678.3828n	723.3810	35.28	M-H, M+FA-H,	C33H58O14	44.90	26.20	0.20	279.2328, 59.0113, 7	########	Gingerglycolipid c	ganjiang
172	36.07_472.3545n	471.3472	36.07	M-H, M+FA-H,	C30H48O4	44.10	24.00	-1.63	471.3474	8333692	Scutellaric acid	baishao
173	37.02_226.2293n	271.2275	37.02	M-H, M+FA-H	C15H30O	39.10	0.03	-1.61	271.2288, 225.2215,	4323289	Pentadecanal	danggui
174	37.02_345.2066n	345.2066	37.02	M-H	C21H30O4	51.20	60.70	-1.46	134.035, 149.0586, 3	4655853	10-dehydrogingerdi	ganjiang
175	37.31_277.2167n	277.2167	37.31	M-H	C18H30O2	39.00	0.00	-2.29	277.2169	########	(z,z,z)-9,12,15-octa	ganjiang
176	37.76_456.3600n	455.3527	37.76	M-H, M+FA-H,	C30H48O3	41.50	9.43	-0.74	78.9563, 152.9936, 3	4873492	Glycyrrhetol	gancao
177	38.86_255.2323n	255.2323	38.86	M-H	C16H32O2	39.00	0.00	-2.53	255.2325	5308992	N-nonanol	danggui,gan
178	39.22_282.2552n	281.2479	39.22	M-H, M+FA-H,	C18H34O2	40.30	6.70	-2.37	281.2484, 80.9624, 4	########	Paradol	ganjiang
179	39.33_571.2884n	571.2884	39.33	M+Na-2H	C30H46O9	38.70	0.11	-0.84	78.9564, 571.2888, 2	########	Ruvoside	gancao
180	40.29_283.2638n	283.2638	40.29	M-H	C18H36O2	39.20	0.00	-1.69	283.2639, 281.2477	2736541	Stearic acid	baishao

181	41.72_576.4387n	621.4369	41.72	M+FA-H, 2M-H	C35H60O6	54.30	76.20	-0.59	1151.8683, 575.4308	2250032	Daucosterin	baishao,dang

Supplementary Figure 2

We performed UPLC-MS for WMP, and the marker compounds in each of the herbal medicines composing WMP were similarly examined. Comparative analyses were also performed.

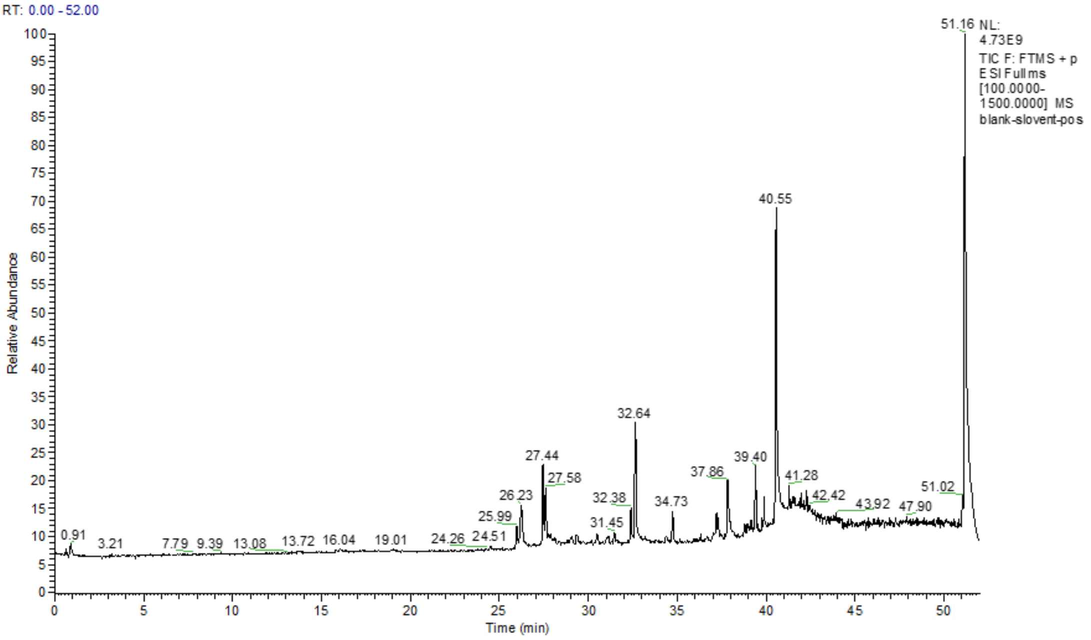

Fig.1. TIC diagram of solvent cation

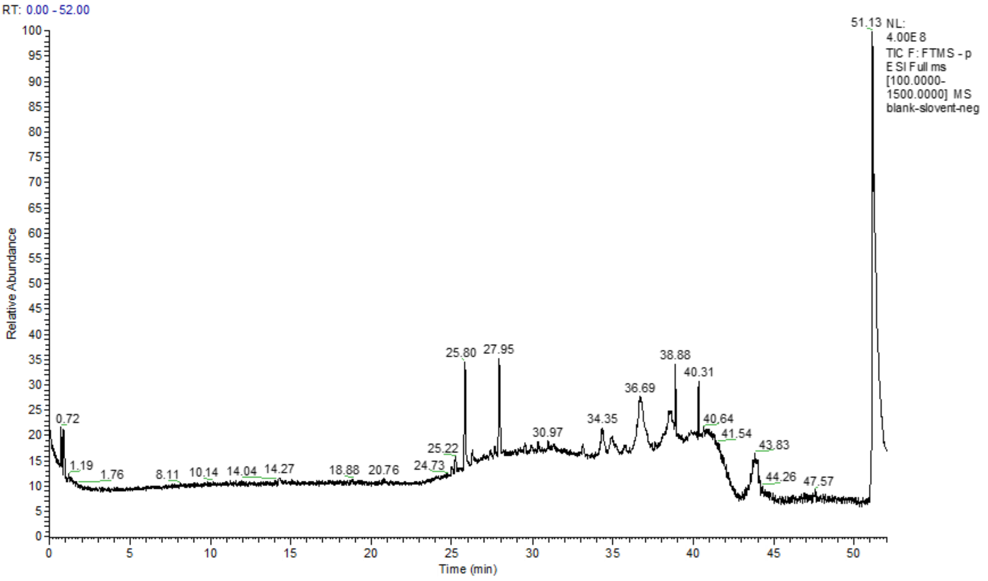

Fig.2. TIC diagram of negative ion

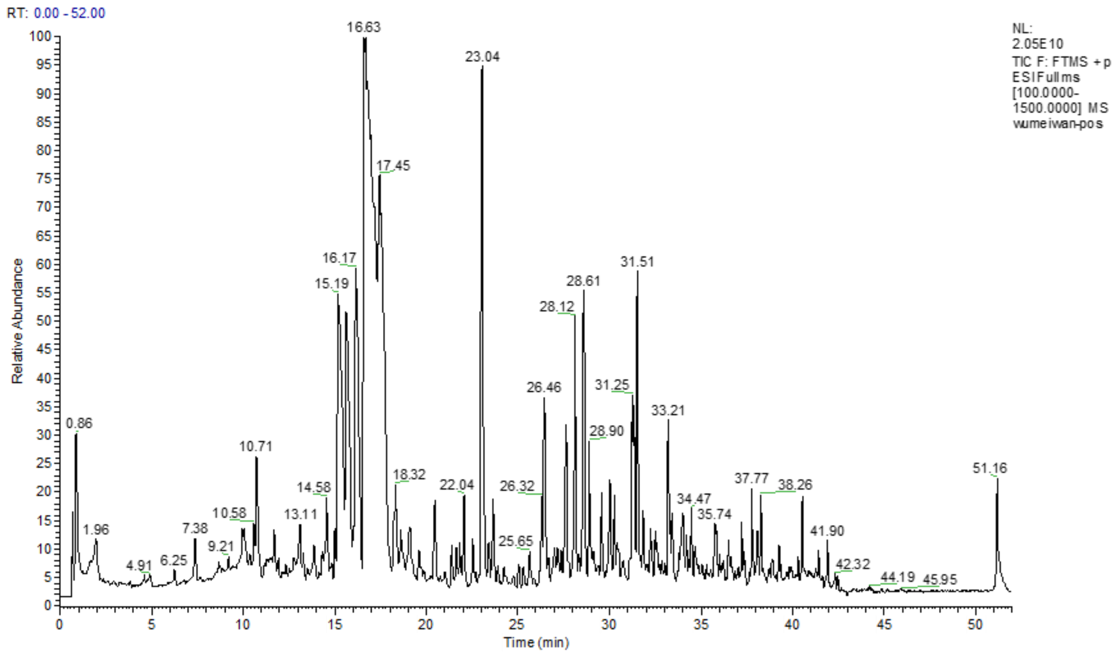

Fig.3.WMP cation TIC plots

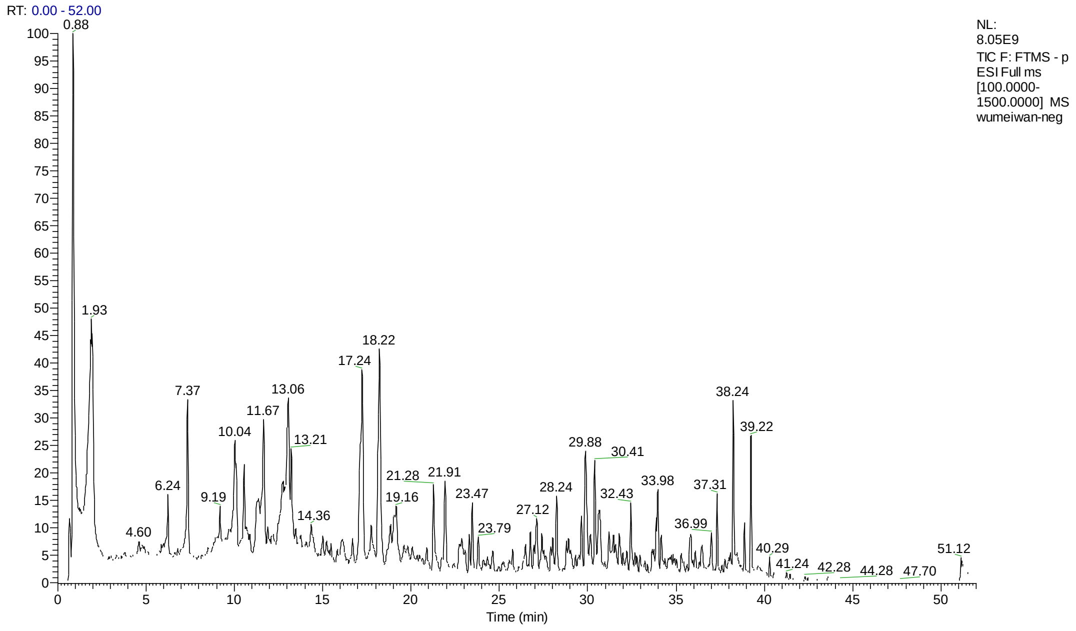

Fig.4.WMP negative ion TIC diagram.

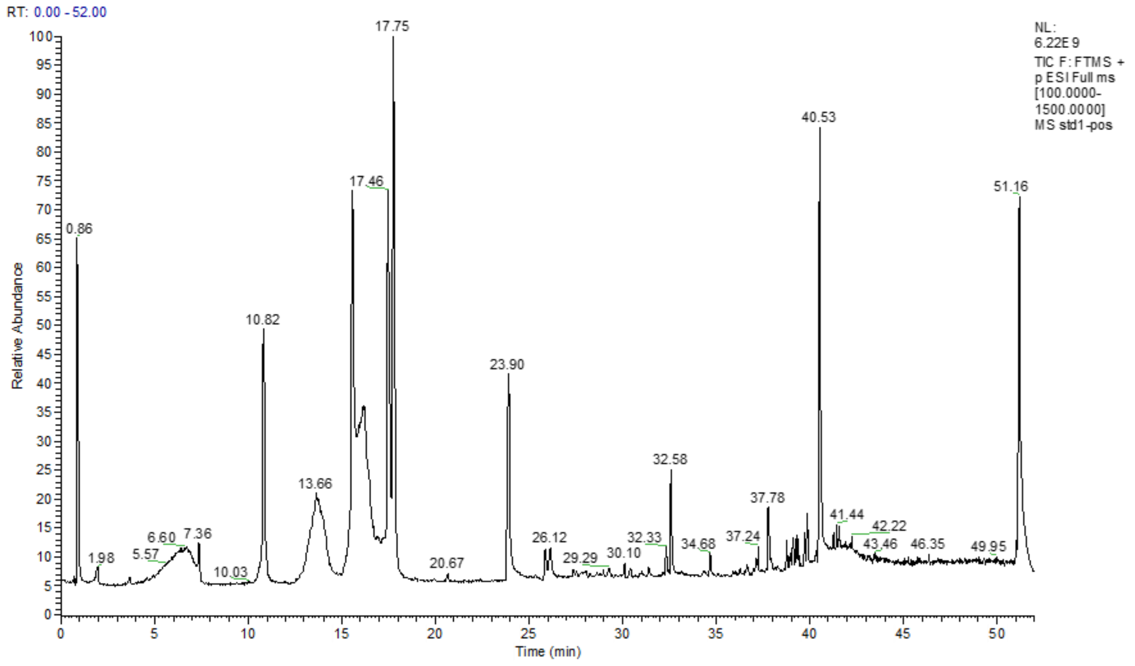

Fig.5.Standard 1 positive ion TIC diagram

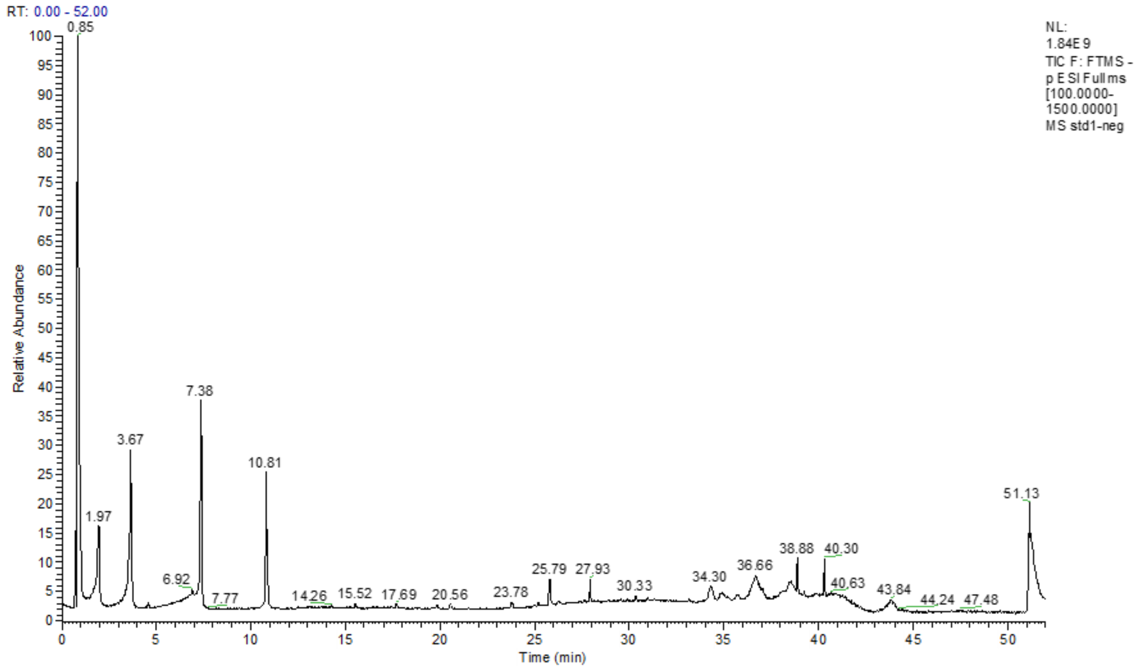

Fig.6.Standard 1 Negative Ion TIC Chart

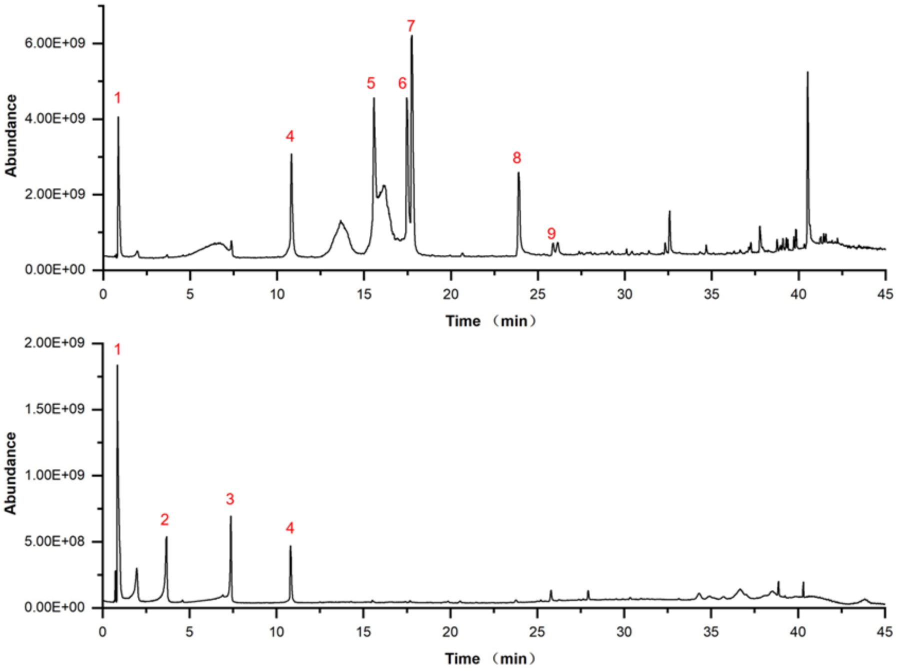

Fig.7. Compositional designation of Reference Standard 1 (1. citric acid 2. gallic acid 3. neochlorogenic acid 4. magnoflorine 5. xanthophylline 6. berberine 7. palmatine 8. cacodyl alcohol 9. methyl eugenol)

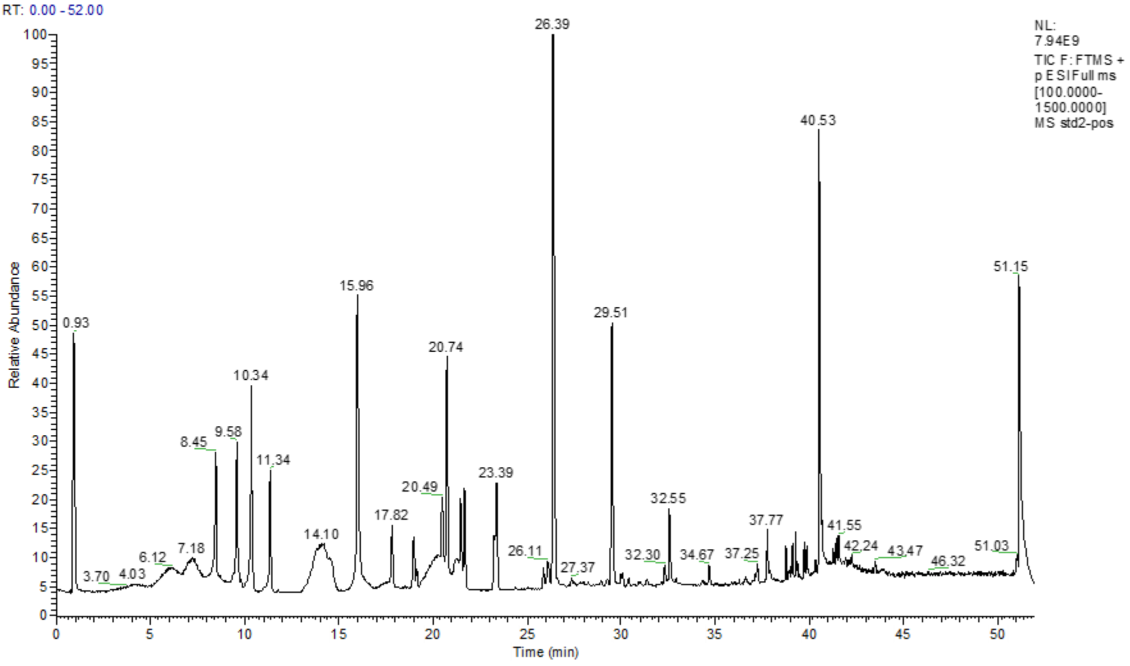

Fig.8.Positive ion TIC diagram of standard 2

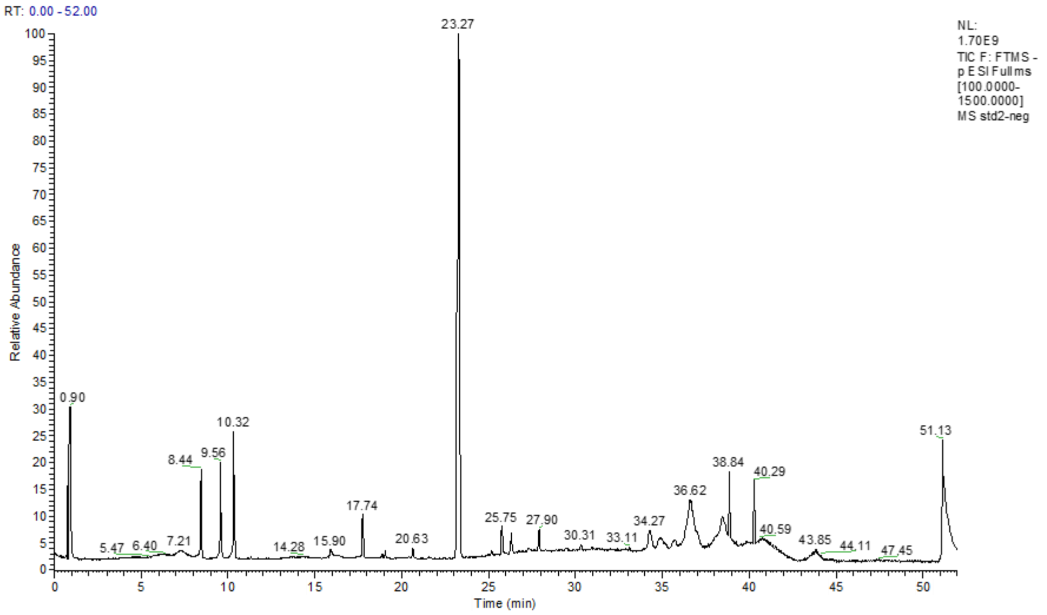

Fig.9.Negative ion TIC diagram of standard 2

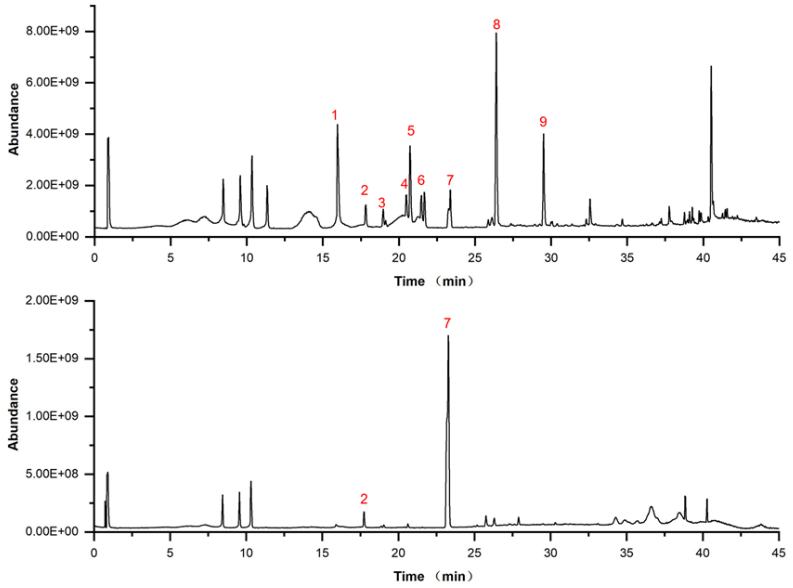

Fig.10. Component identification of standard 2 (1. epiberberine 2. benzoyl neoaconitine 3. benzoyl hypaconitine 4. benzoylaconitine 5. neoaconitine 6. hypaconitine 7. ginsenoside Re 8. 6-gingerol 9. sphingolipids)

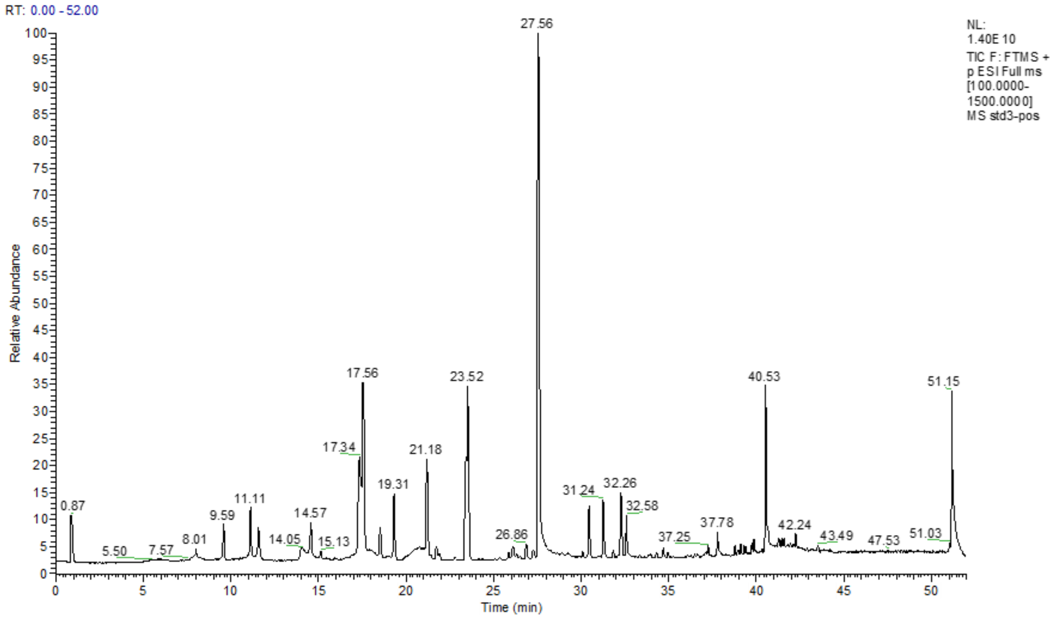

Fig.10. Standard 3 positive ion TIC diagram

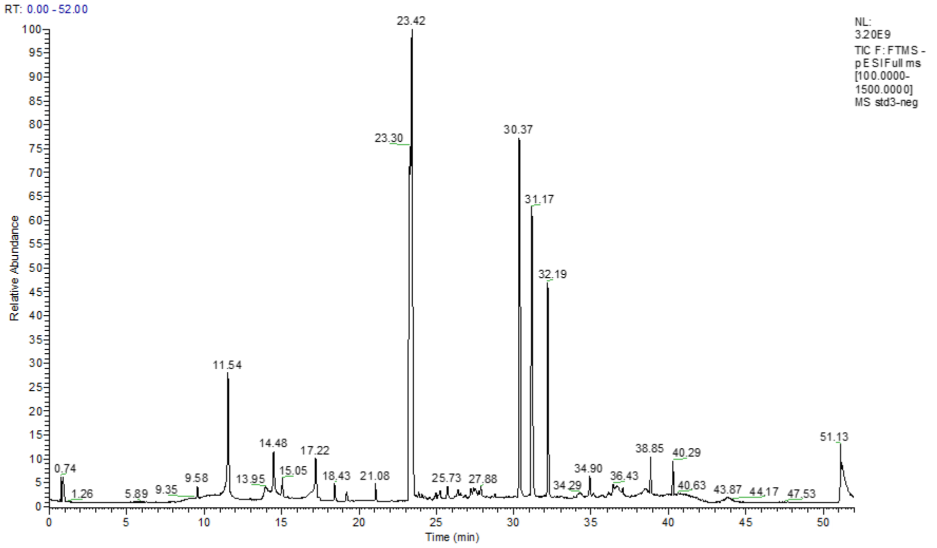

Fig.11. Standard 3 Negative Ion TIC Chart

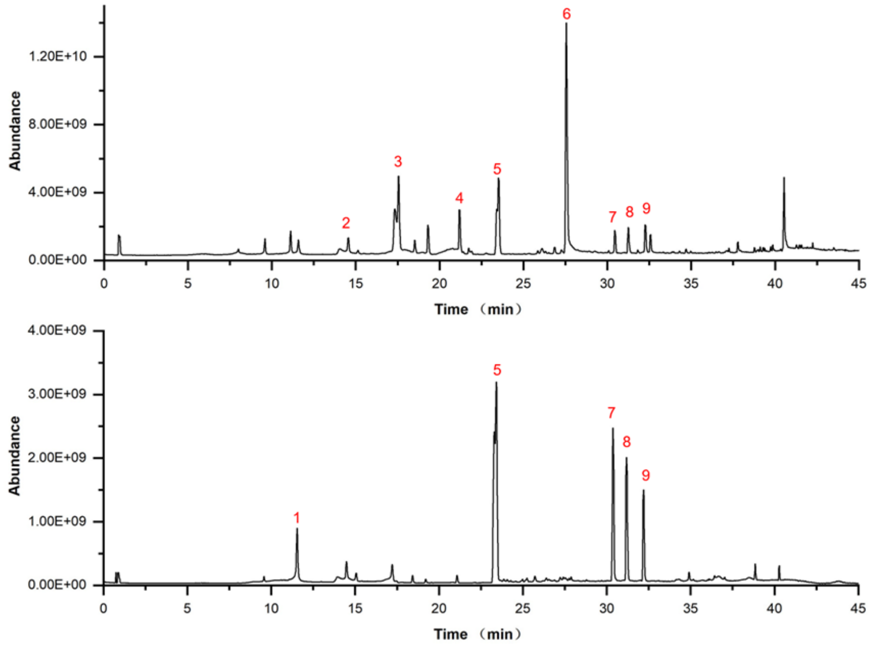

Fig.12. Component identification of Reference Standard 3 (1. Paeonia lactone glycoside 2. ferulic acid 3. rhizoma ligusticum lactone I 4. aconitine 5. ginsenoside Rg1 6. gibberellin 7. ginsenoside Rb1 8. ginsenoside Rb2 9. ginsenoside Rd)

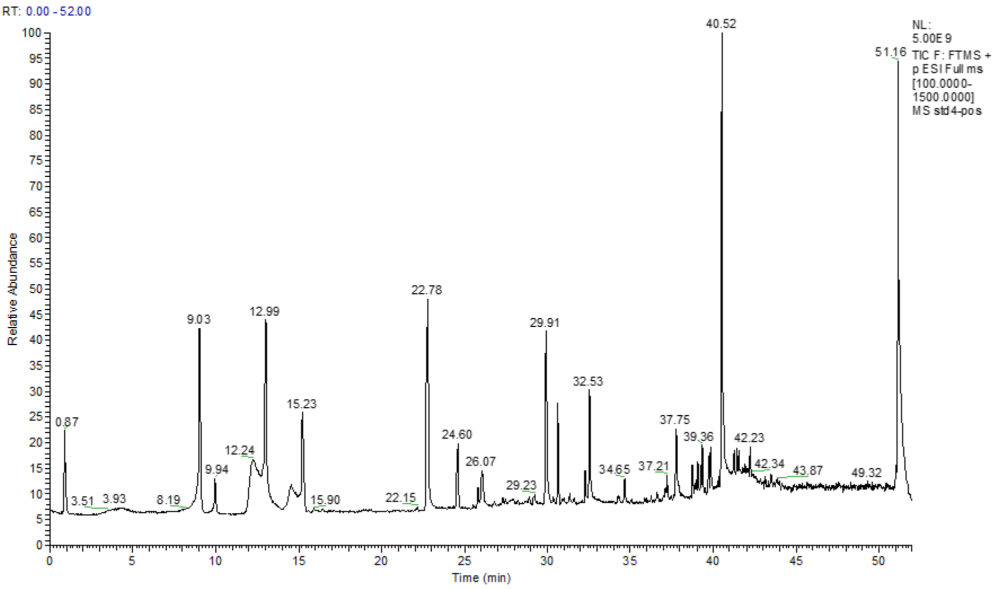

Fig.13. Standard 4 positive ion TIC diagram

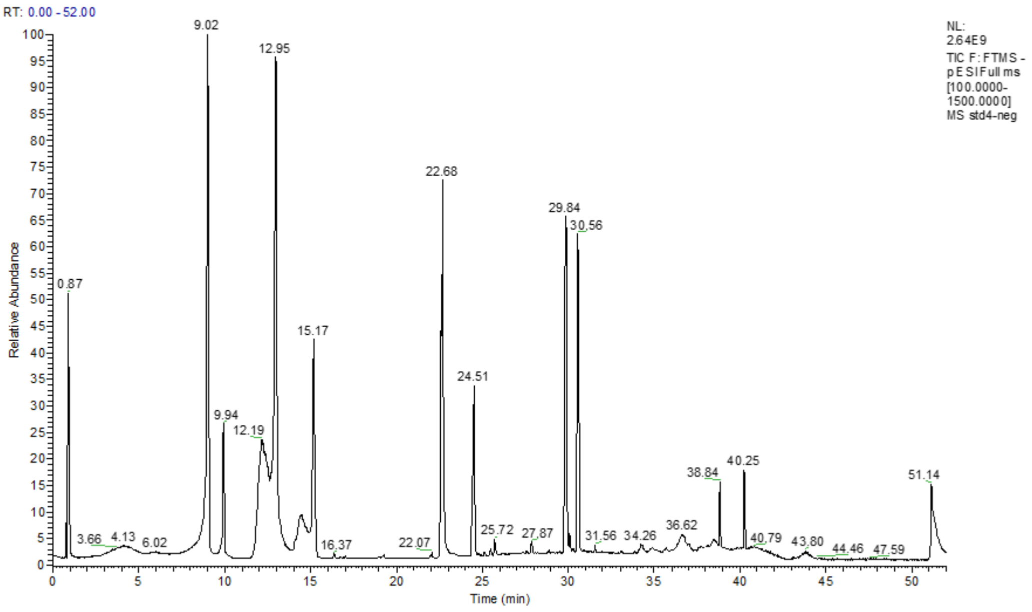

Fig.14.Standard 4 Negative Ion TIC Chart

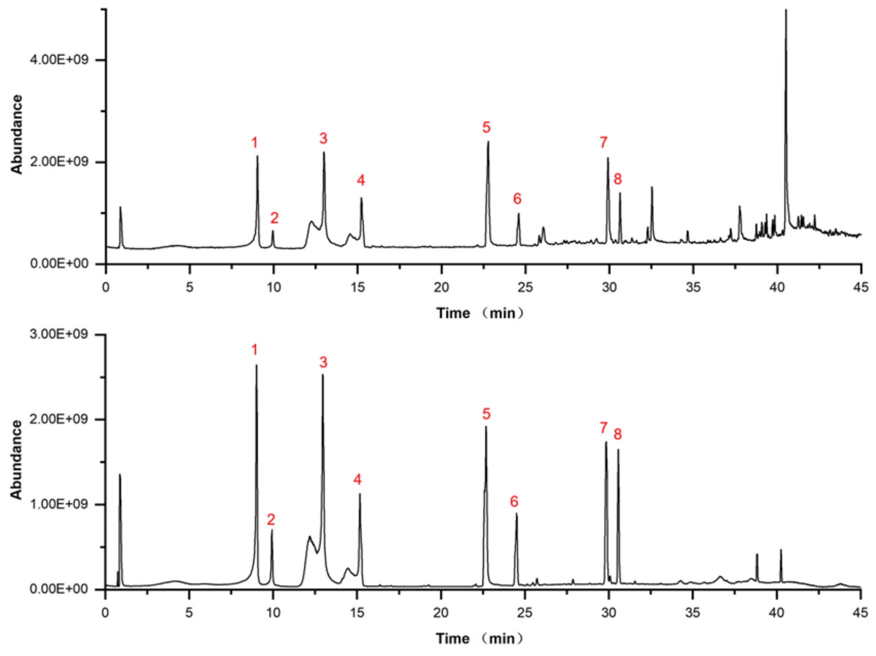

Fig.15. Component identification of Reference Standard 4 (1. catechin 2. paeoniflorin oxide 3. paeoniflorin 4. glycyrrhizin 5. benzoylpaeoniflorin 6. platycodin D 7. glycyrrhizic acid 8. ginsenoside Rc)

Supplementary Figure 3

Rat fibroblast-like synovial cells (RA-FLS)

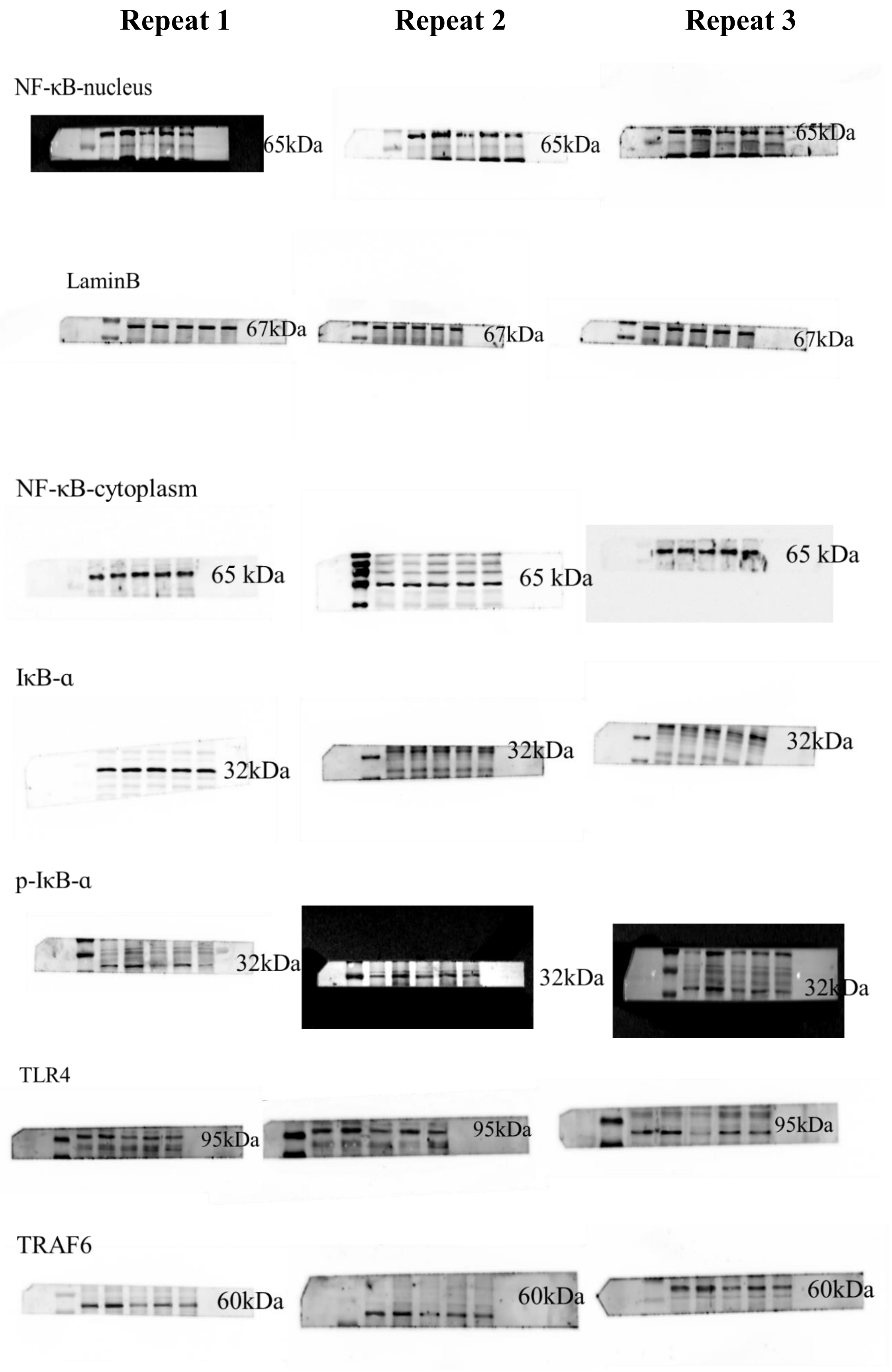

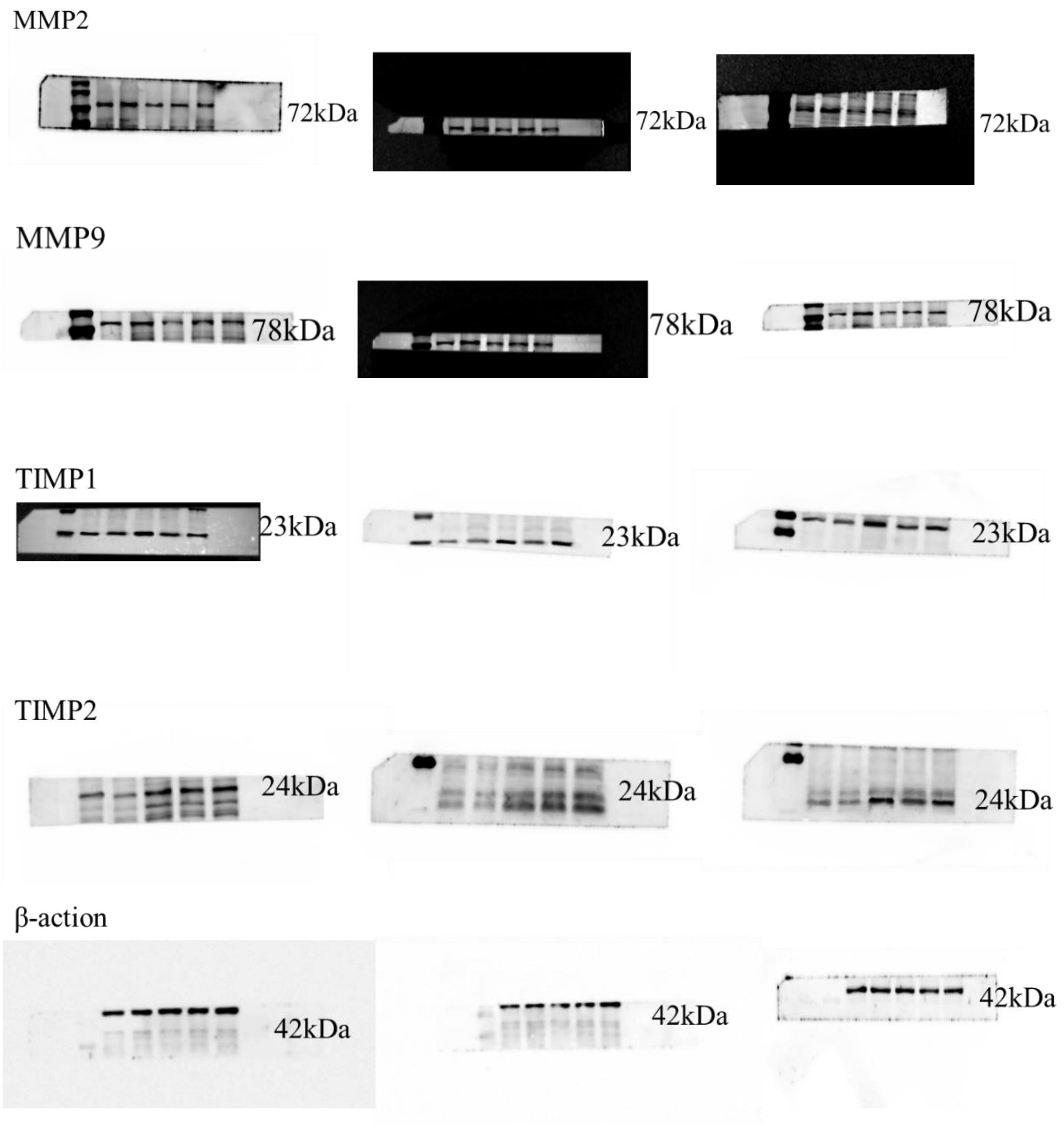
MMP2
72kDa
72kDa
72kDa
MMP9
78kDa
78kDa
78kDa
TIMP1
23kDa
23kDa
23kDa
TIMP2
24kDa
24kDa
24kDa
β-action
42kDa
42kDa
42kDa

Rat synovial tissue

Repeat 1 **Repeat 2** **Repeat 3**

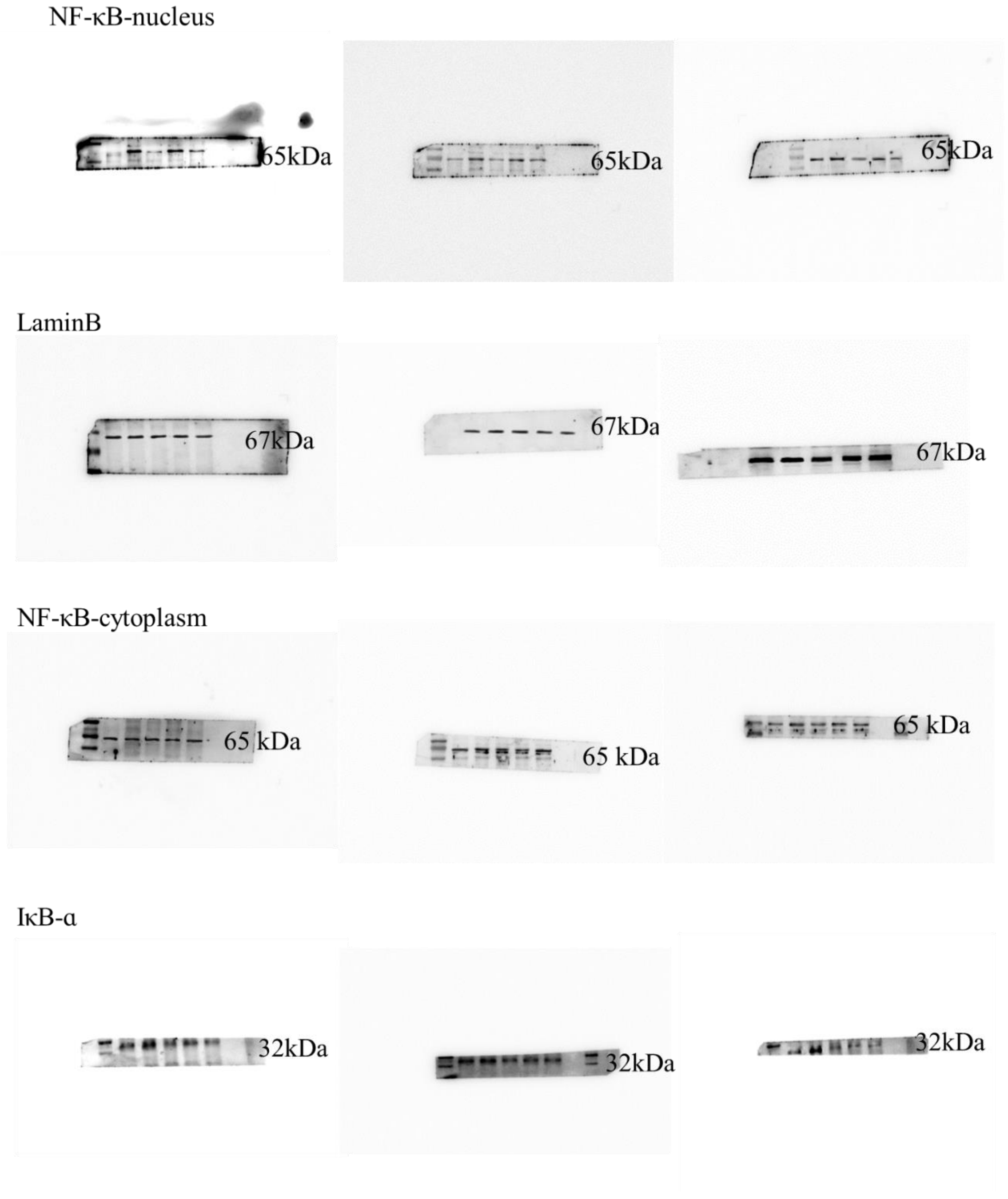

p-IκB-α

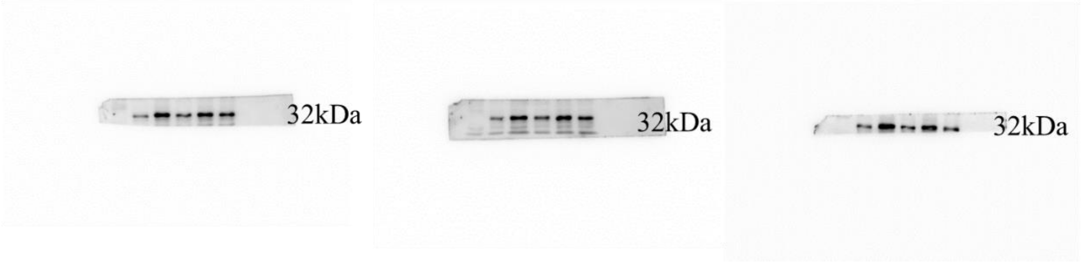

TLR4

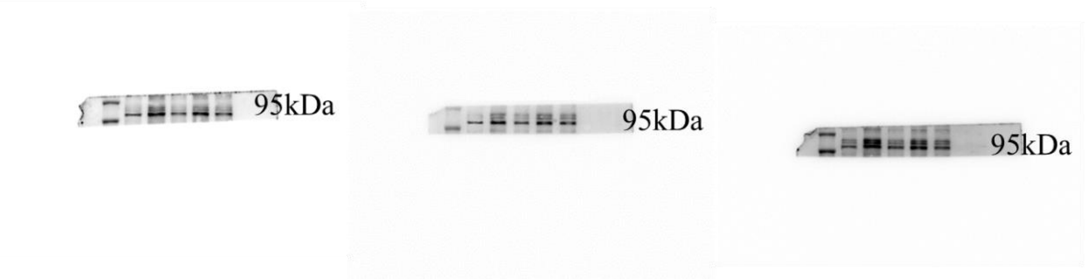

TRAF6

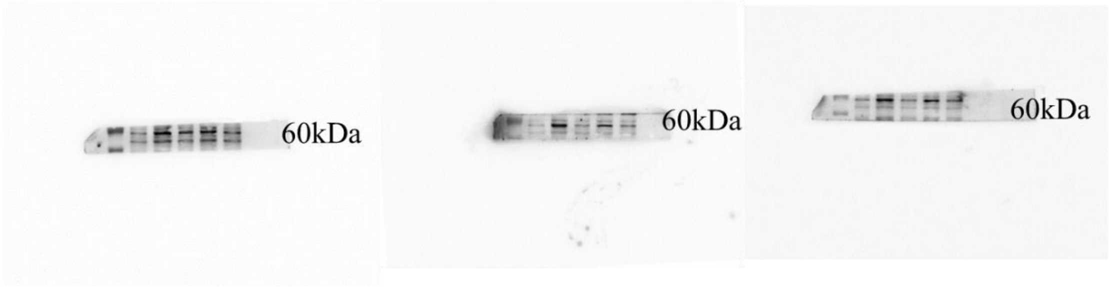

MMP2

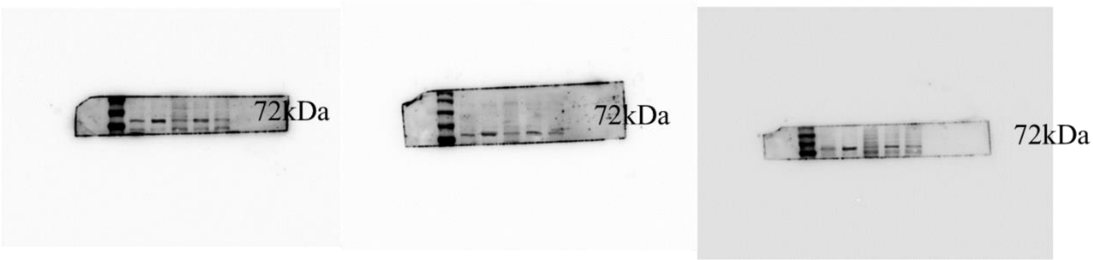

MMP9

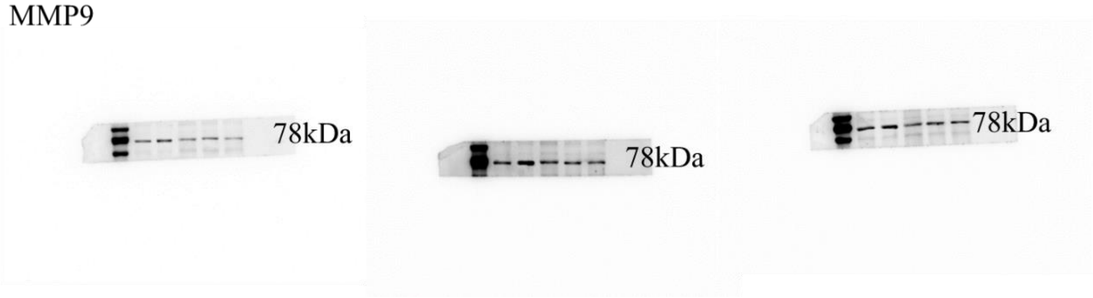

TIMP1

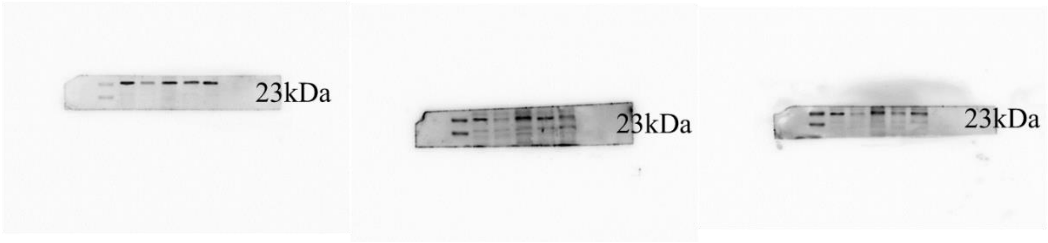

TIMP2

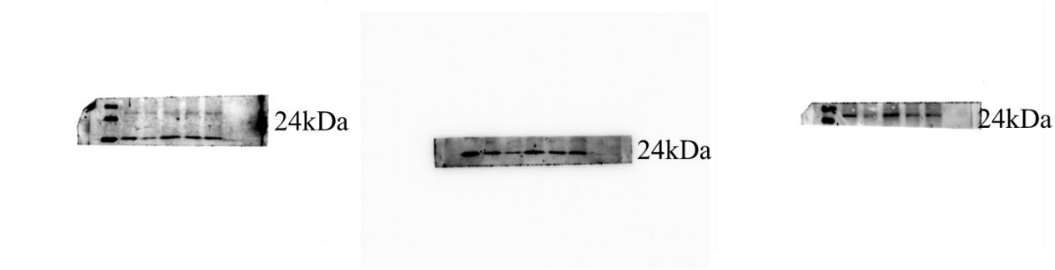

β-action

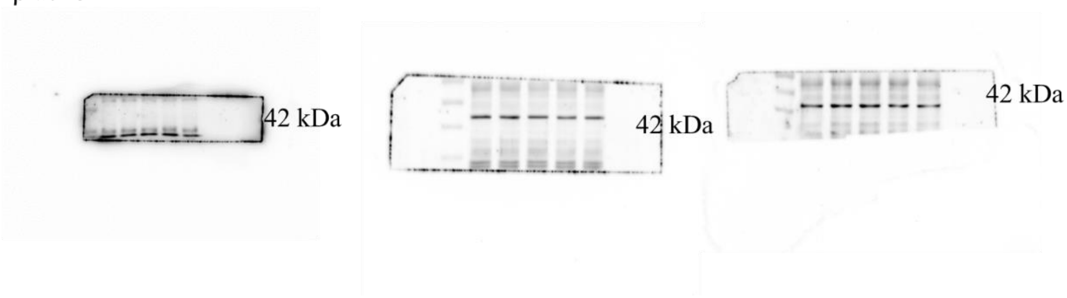

Supplement: Supplementary file 1 — Additional file 1. WMP composition analysis and western blot of the original image. [file 13018_2024_4551_MOESM1_ESM.pdf]
